# Single-Molecule Imaging Reveals Rapid Estradiol Action on the Surface Movement of AMPA Receptors in Live Neurons

**DOI:** 10.3389/fcell.2021.708715

**Published:** 2021-09-23

**Authors:** Soma Godó, Klaudia Barabás, Ferenc Lengyel, Dávid Ernszt, Tamás Kovács, Miklós Kecskés, Csaba Varga, Tibor Z. Jánosi, Géza Makkai, Gergely Kovács, Barbara Orsolits, Takahiro Fujiwara, Akihiro Kusumi, István M. Ábrahám

**Affiliations:** ^1^PTE-NAP Molecular Neuroendocrinology Research Group, Centre for Neuroscience, Szentágothai Research Center, Medical School, Institute of Physiology, University of Pécs, Pécs, Hungary; ^2^PTE-NAP Cortical Microcircuits Research Group, Institute of Physiology, Medical School, Centre for Neuroscience, Szentágothai Research Institute, Pécs, Hungary; ^3^Laboratory of Neuroimmunology, Institute of Experimental Medicine of the Hungarian Academy of Sciences, Budapest, Hungary; ^4^Institute for Integrated Cell-Material Sciences (WPI-iCeMS), Kyoto University, Kyoto, Japan; ^5^Membrane Cooperativity Unit, Okinawa Institute of Science and Technology Graduate University (OIST), Onna, Japan

**Keywords:** 17β-estradiol, AMPAR, single-molecule tracking, diffusion, synapse

## Abstract

Gonadal steroid 17β-estradiol (E2) exerts rapid, non-genomic effects on neurons and strictly regulates learning and memory through altering glutamatergic neurotransmission and synaptic plasticity. However, its non-genomic effects on AMPARs are not well understood. Here, we analyzed the rapid effect of E2 on AMPARs using single-molecule tracking and super-resolution imaging techniques. We found that E2 rapidly decreased the surface movement of AMPAR via membrane G protein-coupled estrogen receptor 1 (GPER1) in neurites in a dose-dependent manner. The cortical actin network played a pivotal role in the GPER1 mediated effects of E2 on the surface mobility of AMPAR. E2 also decreased the surface movement of AMPAR both in synaptic and extrasynaptic regions on neurites and increased the synaptic dwell time of AMPARs. Our results provide evidence for understanding E2 action on neuronal plasticity and glutamatergic neurotransmission at the molecular level.

## Introduction

The gonadal steroid, 17β-estradiol (E2), plays a role in a wide range of biological functions, from fertility to neuroprotection (McEwen, 2002; Kwakowsky et al., 2013; Marbouti et al., 2020a,b; Hokenson et al., 2021). The cellular effects of E2 have been proposed to be mediated by a slow transcriptional action through the nuclear receptors, ERα, and ERβ. In addition to its classical genomic effects, E2 exerts non-classical actions. It rapidly alters the function of receptors and the activity of second messengers through membrane estrogen receptors, such as membrane-associated ERα and ERβ, as well as the G protein-coupled estrogen receptor 1 (GPER1) (Rudolph et al., 2016).

Glutamatergic neurotransmission and synaptic plasticity are also promptly regulated by E2 (Teyler et al., 1980; Wong and Moss, 1992; Kramár et al., 2009b; Ledoux et al., 2009; Vierk et al., 2014; Murakami et al., 2018; Lu et al., 2019). Extracellularly recorded dendritic field potentials in the hippocampal CA1 subfield and miniature excitatory synaptic currents (mEPSCs) recorded via whole-cell voltage-clamp in the CA1 pyramidal cells of adult rats are rapidly altered by E2 (Phan et al., 2015; Oberlander and Woolley, 2016). However, the E2 effect is selective to a subset of neurons and the molecular mechanism differs between sexes probably due to the different ER profile which can lead to different effect on neurons (Wong and Moss, 1992).

The surface movement of glutamate receptors, such as AMPARs, is crucial in excitatory neurotransmission and synaptic plasticity (Babayan and Kramár, 2013; Penn et al., 2017). The submembrane actin network affects excitatory neurotransmission and surface movement of AMPARs (Kramár et al., 2006; Gowrishankar et al., 2012). The amount, distribution, and movement of AMPAR molecules in the postsynaptic density and the extrasynaptic sites determine the efficiency and function of the synapse (Ashby et al., 2004; Groc and Choquet, 2006; Lee et al., 2017; Choquet, 2018). Steroid hormones such as corticosterone and aldosterone, have been shown to rapidly alter the membrane dynamics of AMPARs, as well as the synaptic dwell time (the time spent within the active site of synapse) (Groc et al., 2008). However, it is unknown whether E2 affects the surface movement of AMPARs. We applied E2 to live neurons and performed multiple super-resolution imaging and single-molecule tracking approaches to examine the effects of E2 on the surface movement of glutamate receptor molecules. Our findings demonstrated that E2 rapidly decreased the surface movements of GluR2-AMPAR molecules [the most abundant AMPAR subunit in neurons (Isaac et al., 2007)] in a dose-dependent manner without affecting mGluR1 molecules [a metabotropic glutamate receptor 1 involved in the rapid membrane action of E2 (Micevych and Mermelstein, 2008)] in neuronal cells differentiated from rat pheochromocytoma (PC12) cells (dPC12). The mechanism of the E2 action is compartment-specific and is mediated by ER mechanisms involving the cortical actin and cofilin pathways. Our results gained from dPC12 were confirmed by cultured hippocampal neurons, a more differentiated system with mature synapses. In hippocampal neurons E2 also decreased the surface movements of GluR2-AMPAR. This study provides the first evidence that E2 decreases the surface movement of synaptic GluR2-AMPAR and increases the dwell time of GluR2-AMPAR in the synapse. These findings broaden our knowledge of the molecular mechanism of E2 action on neuronal plasticity and glutamatergic neurotransmission.

## Results

### Characterization of Neuronal Properties of dPC12 and Single-Molecule Tracking of ATTO 488-Labeled GluR2-AMPAR and mGluR1

We characterized the PC12 cells after 4 days of NGF treatment when neurite outgrowth was pronounced (Supplementary Figure 1A). Immunocytochemistry showed that dPC12 expressed neuronal markers such as β-III tubulin and MAP-2 (Supplementary Figure 1B). In addition, we examined the passive electrophysiological parameters of 10 cells using whole-cell patch clamp technique. We found that the resting membrane potential, the input resistance and the cell capacitance were −55.5 ± 7.7 mV, 1072.7 ± 854.9 MΩ and 60.2 ± 32.9 pF, respectively (values are represented as mean ± SD). Finally, we recorded that step current injection elicited a single abortive action potential in dPC12 (Supplementary Figure 1C). Moreover, *in vivo* labeling of dPC12 demonstrated GluR2-AMPAR and mGluR1 in neurites and soma (Supplementary Movies 1–4).

**FIGURE 1:**
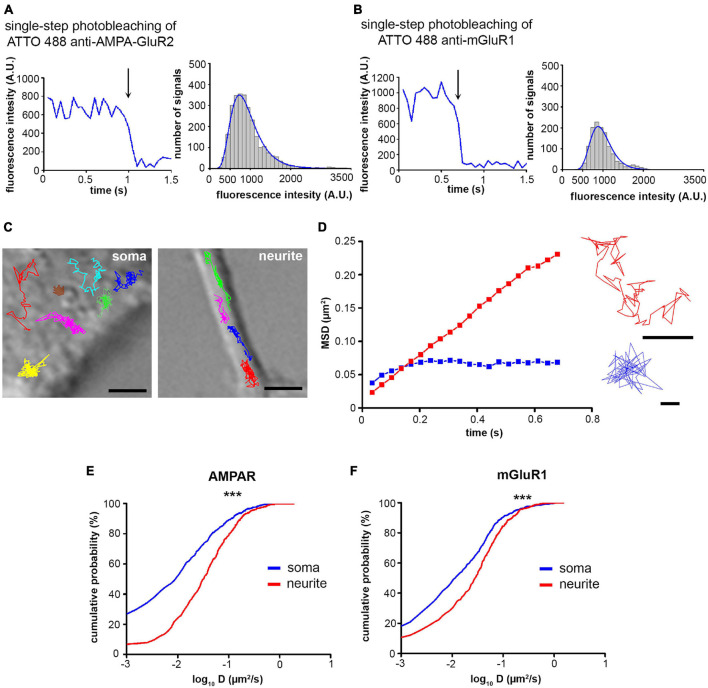
Characterization of differentiated PC12 cells and validation of single-molecule labeling. **(A,B)** Left, Intensity profiles of a single ATTO 488-labeled GluR2-AMPAR **(A)** and mGluR1 **(B)** signal. The arrows indicate single-step photobleaching. Right, Histogram showing the intensity value of every spot found in a recording of ATTO 488-labeled GluR2-AMPAR **(A)** and mGluR1 **(B)**, superimposed with a single fitted lognormal curve (blue line). **(C)** Representative trajectories of AMPAR molecules on somas and neurites. Scale bar = 2 μm. **(D)** The mean square displacement functions and trajectories represent AMPAR molecules with Brownian motion (red) and confined motion (blue). Scale bar = 0.1 μm. **(E,F)** The cumulative probability functions of D values of AMPAR **(E)** and mGluR1 **(F)** on neurites and somas (*n* = 510–676 trajectories). ***p < 0.001.

In single-molecule tracking experiments, the fluorescence intensity versus time function showed one-step photobleaching, representing single ATTO 488 fluorophores for GluR2-AMPAR and mGluR1. The fluorescence intensity histograms of both GluR2-AMPAR and mGluR1 had peak intensities similar to those of the step sizes for photobleaching (Figures 1A,B). These results suggest that most of the spots represented single fluorophores and single receptors.

### E2 Rapidly Decreases the Surface Movement of GluR2-AMPAR Molecules in dPC12

#### Surface Movements of GluR2-AMPARs and mGluR1 on dPC12

The surface movement of glutamate receptors was detected in the plasma membrane of live dPC12 (Figure 1C and Supplementary Movies 1–4). Based on the mean square displacement functions of GluR2-AMPARs and mGluR1 receptors, they exhibited two main types of movements: Brownian diffusion, when receptors moved freely between barriers and confined motion when receptors were restricted to a small area (Figure 1D). The diffusion coefficients of both receptors are significantly higher on the neurite than on soma (Figures 1E,F), indicating that the surface movement of glutamate receptors is faster on neurites.

#### Dose Dependence

**FIGURE 2:**
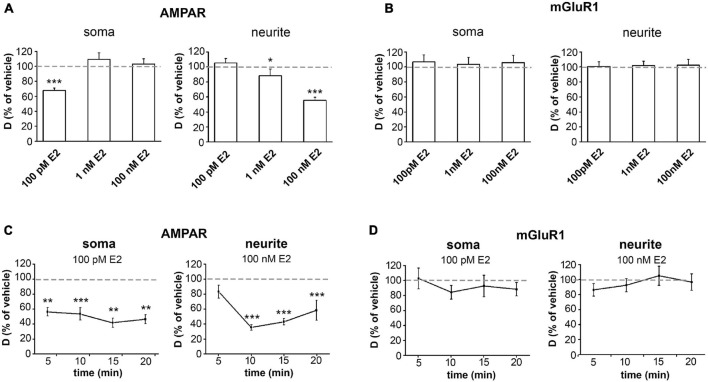
Effect of E2 on the surface movement of GluR2-AMPAR and mGluR1. **(A)** Effect of different concentrations of E2 on the diffusion coefficient (D, μm^2^/s) of GluR2-AMPAR **(A)** and mGluR1 **(B)** (% of vehicle treatment as the mean ± SEM, *n* = 425–1145 trajectories per group). **(C,D)** Line graphs depict changes in D of GluR2-AMPAR **(C)** and mGluR1 **(D)** molecules at different time points after the administration of the most effective concentration of E2 (% of vehicle treatment as the mean D ± SEM, *n* = 117–187 trajectories per time point). *p < 0.05; **p < 0.01; ***p < 0.001.

Administration of 100pM, 1 nM and 100 nM doses of E2 evoked a clear dose-dependent decrease in D_AMPAR_ in neurites as measured in the first 20 min after treatment with a maximum decrease of 55% (*p* < 0.01) (vehicle mean D_AMPAR_ ± SEM (μm^2^/s) on neurite: 0.058 ± 0.003) (Figure 2A and Supplementary Movie 5). In soma, 100 pM of E2 significantly decreased D_AMPAR_ (68%, *p* < 0.01), while 1 nM and 100 nM of E2 were ineffective (vehicle mean D_AMPAR_ ± SEM [μm^2^/s] on soma: 0.024 ± 0.002) (Figure 2A). In contrast, E2 (100 nM, 1 nM or 100 pM) did not change D_mGluR1_ either in soma or in neurites (Figure 2B) (vehicle mean D_mGluR1_ ± SEM (μm^2^/s); soma: 0.032 ± 0.003, neurite: 0.049 ± 0.005).

To investigate whether a low concentration of EtOH (10^–3^ %) (vehicle) affects GluR2-AMPAR and mGluR1 surface trafficking, we compared D_AMPAR_ and D_mGluR1_ in a culture medium (control) without or with vehicle (20 min after application). There was no significant effect of vehicle on D_AMPAR_ and D_mGluR1_ in dPC12 [values are expressed as the mean D ± SEM [μm^2^/s]; on soma: control D_AMPAR_: 0.024 ± 0.003 (*n* = 590 trajectories), vehicle D_AMPAR_: 0.022 ± 0.002 (*n* = 612 trajectories); neurite: control D_AMPAR_: 0.073 ± 0.006 (*n* = 545 trajectories), vehicle D_AMPAR_: 0.069 ± 0.007, (*n* = 647 trajectories); soma: control D_mGluR1_: 0.033 ± 0.003 (*n* = 751 trajectories), vehicle D_mGluR1_: 0.034 ± 0.002, (*n* = 622 trajectories); neurite: control D_mGluR1_: 0.051 ± 0.004 (*n* = 513 trajectories), vehicle: 0.050 ± 0.003, (*n* = 496 trajectories)].

#### Time Course

To examine the time dependence of the effect evoked by E2 on D_AMPAR_ or D_mGluR1_, we applied the most effective E2 doses on soma and neurites and measured D at different time points. The application of 100 pM of E2 resulted in a significant decrease (*p* < 0.01) in D_AMPAR_ on soma within 5 min. This remained reduced at 10 min, 15 min, and 20 min (vehicle mean D_AMPAR_ ± SEM (μm^2^/s) on soma: 5 min, 0.064 ± 0.007; 10 min, 0.054 ± 0.008; 15 min, 0.03 ± 0.004; 20 min, 0.042 ± 0.008). In contrast, 100 nM of E2 only reduced D_AMPAR_ on neurites at 10, 15, and 20 min (vehicle mean D_AMPAR_ ± SEM [μm^2^/s] on neurites: 5 min: 0.063 ± 0.007; 10 min: 0.051 ± 0.005; 15 min: 0.050 ± 0.007; 20 min: 0.051 ± 0.007) (Figure 2C). In contrast, 100 pM or 100 nM of E2 did not affect D_mGluR1_ on neurites or soma, respectively, at any time point (vehicle mean D_mGluR1_ ± SEM [μm^2^/s] on soma: 5 min: 0.033 ± 0.006; 10 min: 0.042 ± 0.006 15 min: 0.031 ± 0.005; 20 min: 0.036 ± 0.007; on neurites: 5 min: 0.061 ± 0.006; 10 min: 0.053 ± 0.007; 15 min: 0.052 ± 0.004; 20 min: 0.038 ± 0.004) (Figure 2D).

### GPER1 and ERβ Mediate the Effect of E2 on the Surface Movement of GluR2-AMPAR Molecules in dPC12

**FIGURE 3:**
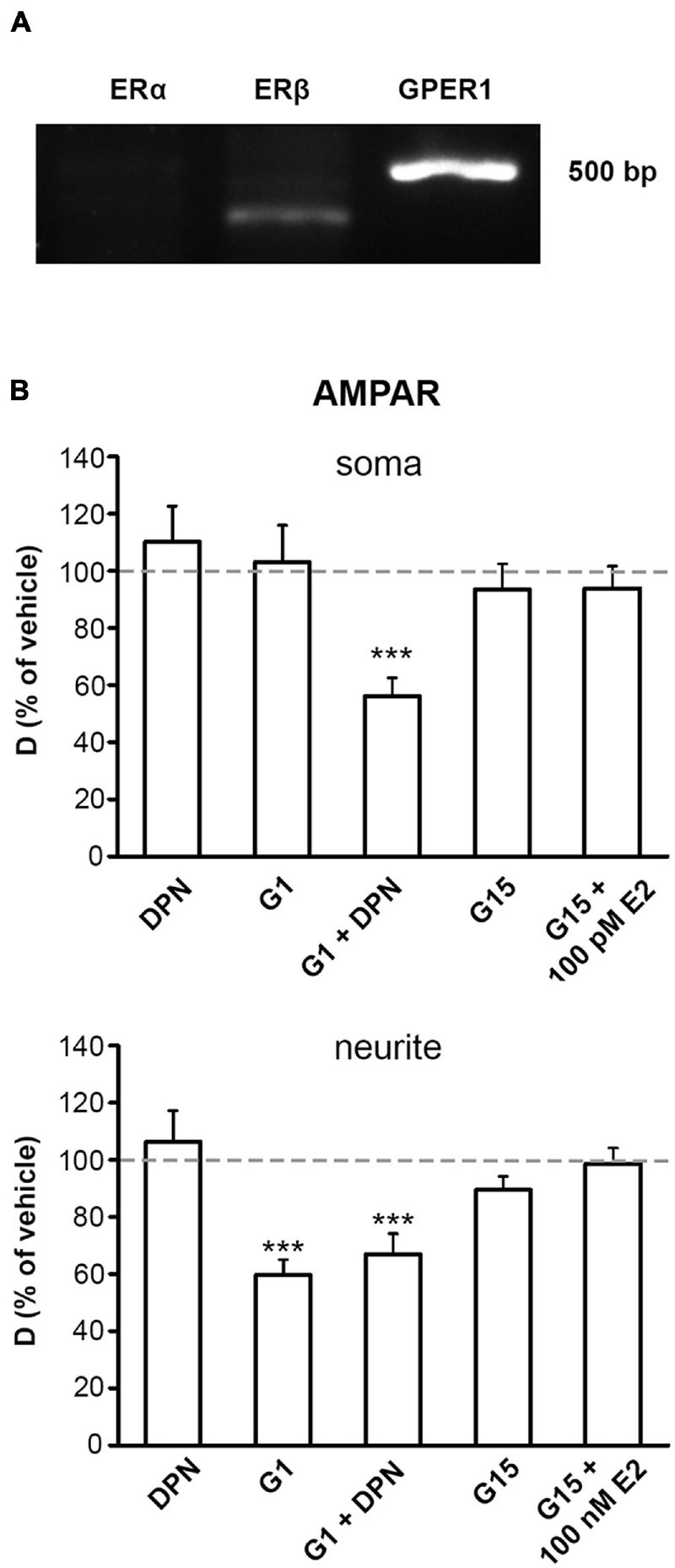
Effect of estrogen receptor modulation on the surface movement of GluR2-AMPAR. **(A)** Representative PCR gel electrophoresis image depicting the expression of estrogen receptor beta (ERβ) and G protein-coupled estrogen receptor 1 (GPER1) mRNA in dPC12. Estrogen receptor alpha (ERα) mRNA was not detected. **(B)** Histograms demonstrate the mean D_AMPAR_ as a percentage of vehicle control on somas and neurites in the presence of the estrogen receptor, β (ERβ) agonist diarylpropionitrile (DPN), a GPER1 agonist (G1), G1+DPN together, a GPER1 antagonist (G15) and G15+E2 (with 100 pM of E2 on the somas and 100 nM of E2 on the neurites) (mean ± SEM; *n* = 215–641 trajectories). ***p < 0.001.

Our PCR results revealed that dPC12 expresses ERβ and GPER1, but not ERα (Figure 3A). Although the addition of ERβ agonist DPN (10 pM) or specific GPER1 agonist G1 (100 nM) alone did not affect the surface movement of somatic GluR2-AMPAR molecules (vehicle mean D_AMPAR_ ± SEM (μm^2^/s) on soma; DPN vehicle: 0.04 ± 0.003; G1 vehicle: 0.023 ± 0.002), co-administration of DPN and G1 decreased D_AMPAR_ (DPN+G1 vehicle D_AMPAR_ mean ± SEM (μm^2^/s) on soma: 0.075 ± 0.009) similar to 100 pM of E2 (Figure 3B). G1 (100 nM) mimicked the effect of 100 nM of E2 without and with 10 pM of DPN (vehicle mean D_AMPAR_ ± SEM (μm^2^/s) on neurite; G1 vehicle: 0.056 ± 0.003; G1+DPN vehicle: 0.1 ± 0.004) in neurites (Figure 3B). However, 10 pM of DPN alone did not alter the D_AMPAR_ in neurites (DPN vehicle mean D_AMPAR_ ± SEM (μm^2^/s) on neurite: 0.056 ± 0.004) (Figure 3B). In addition, prior application of 1 μM of G15 blocked the effect of 100 pM of E2 on soma and 100 nM of E2 on neurites (vehicle mean D_AMPAR_ ± SEM (μm^2^/s); soma: 0.025 ± 0.002, neurite: 0.048 ± 0.003, Figure 3B). G15 application alone did not alter the surface movement of GluR2-AMPAR in either neurites or soma (vehicle mean D_AMPAR_ ± SEM (μm^2^/s); soma: 0.020 ± 0.002, neurite: 0.062 ± 0.004, Figure 3B).

Since we applied DMSO as a vehicle in these experiments, we also tested whether the 0.1 % DMSO alone affected D_AMPAR_. We compared D_AMPA__R_ in a culture medium (control) with or without vehicle (20 min after application). There was no significant effect of DMSO on D_AMPAR_ in dPC12 (values are expressed as the mean D ± SEM [μm^2^/s] on soma: medium D_AMPAR_: 0.024 ± 0.003 (*n* = 590 trajectories), vehicle D_AMPAR_: 0.023 ± 0.002 (*n* = 645 trajectories); on neurite: medium D_AMPAR_: 0.073 ± 0.006 (*n* = 545 trajectories), vehicle D_AMPAR_: 0.062 ± 0.004, (*n* = 524 trajectories).

**FIGURE 4:**
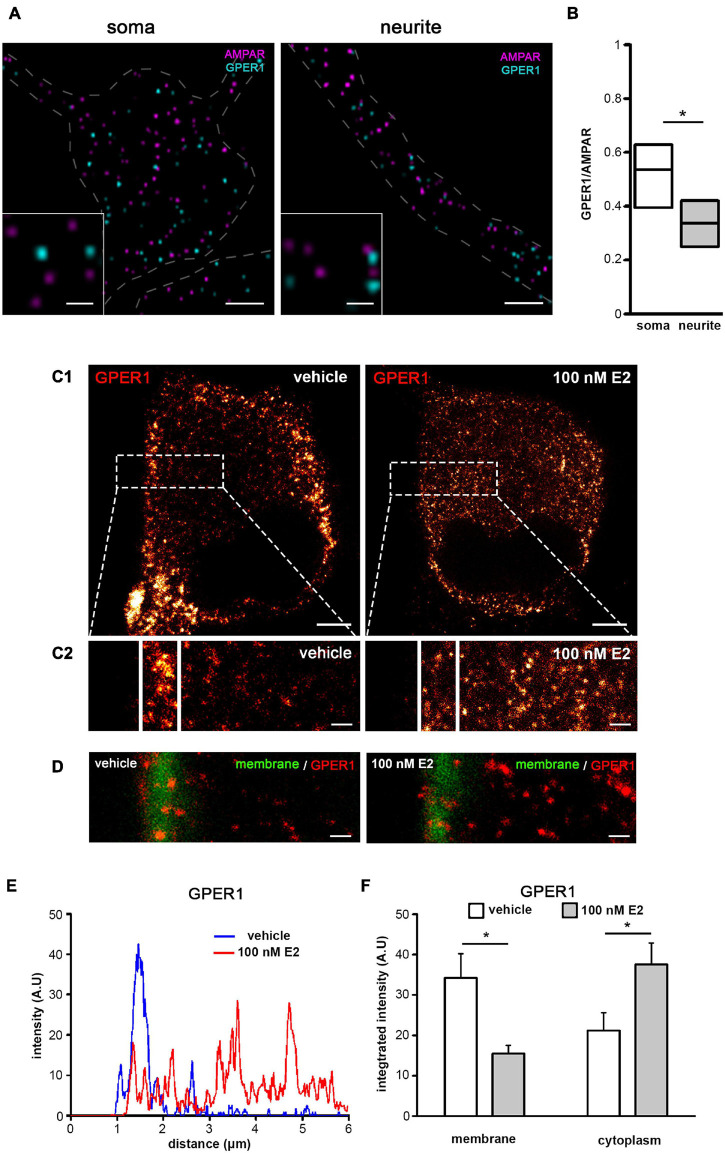
The GluR2-AMPAR/GPER1 ratio and molecular distance between GPER1 and GluR2-AMPAR in the membrane. **(A)** STORM images depicting immunolabeled AMPAR (magenta) and GPER1 (cyan) molecules on dPC12. Dashed lines delineate the borders of the neurites and somas. Scale bar = 2 μm; inset Scale bar = 0.5 μm. **(B)** The ratio between the number of GPER1 and AMPAR molecules (GPER1/GluR2-AMPAR) on the neurites and somas (*n* = 11 somas or neurites). **(C1)** Photomicrographs depict GPER1 immunoreactivity (visualized with STED microscopy) in dPC12 after 10 min of vehicle (left) or of 100 nM of E2 treatment (right). Scale bar = 2 μm. **(C2)** One 2 μm^2^ (between parallel white bars) and one 10 μm^2^ (to the left) areas were selected within each ROI for the membrane and cytoplasmic regions of each cell, respectively. Integrated density was calculated and normalized to the area. Scale bar = 0.5 μm. **(D)** Dual labeling of plasma membrane and GPER1 molecules defines the membrane regions (approximately 1 μm wide). Scale bar = 0.5 μm. **(E)** Line graph of the fluorescent intensity calculated from the magnified STED inserts (C2). **(F)** Integrated density graphs of GPER1 show the effect of vehicle and 100 nM of E2 treatment in the membrane and in the cytoplasm (*n* = 15 cells were evaluated in each group). *p < 0.05.

Our results show that GPER1 mediates the effect of E2 on GluR2-AMPAR on both soma and neurites. To further analyze the relationship between GluR2-AMPAR and GPER1, we used STORM super-resolution imaging to examine the expression GPER1 and GluR2-AMPAR. STORM imaging revealed that GPER1 and GluR2-AMPAR receptors are expressed on both soma and neurites (Figure 4A). In order to examine the number of GPER1 in relation to GluR2-AMPAR we normalized the number of GPER1 to GluR2-AMPAR using GPER1/GluR2-AMPAR ratio. Our analysis demonstrated that the GPER1/GluR2-AMPAR ratio was significantly higher in soma than in neurites of dPC12 (Figure 4B).

E2 can induce rapid internalization and consequent desensitization of GPER1 (Filardo and Thomas, 2012). The internalization of GPER1 may explain the different effects of E2 on the soma and neurites. To visualize whether GPER1 is internalized after E2 administration in soma, stimulated emission depletion (STED) microscopy was used (Figures 4C1,C2). Super-resolution STED imaging revealed that the intensity of immunostaining of GPER1 was approximately 2 times higher in the membrane region than in the cytoplasm of vehicle-treated dPC12 (Figures 4C1,C2,D). After 10 min of 100 nM of E2 treatment, the intensity profile of GPER1 showed a significant decrease in the membrane region (Figures 4C1,C2,D,E). In contrast, the majority of GPER1 immunoreactivity was located in the cytoplasm (Figures 4C1,C2,D,E) after treatment with 100 nM of E2, suggesting rapid internalization of GPER1 in response to high E2 exposure. There was no internalization of GPER1 observed in neurites after 100 nM of E2 treatment (Supplementary Figure 2).

### Critical Role of the Cortical Actin Network in the Effect of E2 on GluR2-AMPAR in dPC12

Cortical actin is a thin actin network that lies directly underneath the plasma membrane. The cortical actin network is essential in the organization of neuronal compartments and plays a crucial role in membrane receptor movement (Schevzov et al., 2012), thus we speculated that the cortical actin network may play a pivotal role in the effect of E2 on the receptor dynamics. Previous studies show that E2 induces cytoskeleton assembly mediated by GPER1 receptors via different intracellular signaling pathways, including the Rho-associated protein kinase (ROCK)-cofilin (Gowrishankar et al., 2012; Wang et al., 2019) and c-Jun-N-terminal kinase (JNK)-cofilin (Kim et al., 2019) pathways. To determine the possible role of cortical actin in the effects of E2 on glutamate receptors, we treated cells with the actin polymerization inhibitor, latrunculinA (latA; l μM). To examine the role of the ROCK-cofilin and JNK-cofilin pathways in E2 action, we applied the ROCK inhibitor, GSK429286 (l μM) (Wang et al., 2019), and JNK inhibitor, SP600125 (l μM) (Kim et al., 2019), respectively.

**FIGURE 5:**
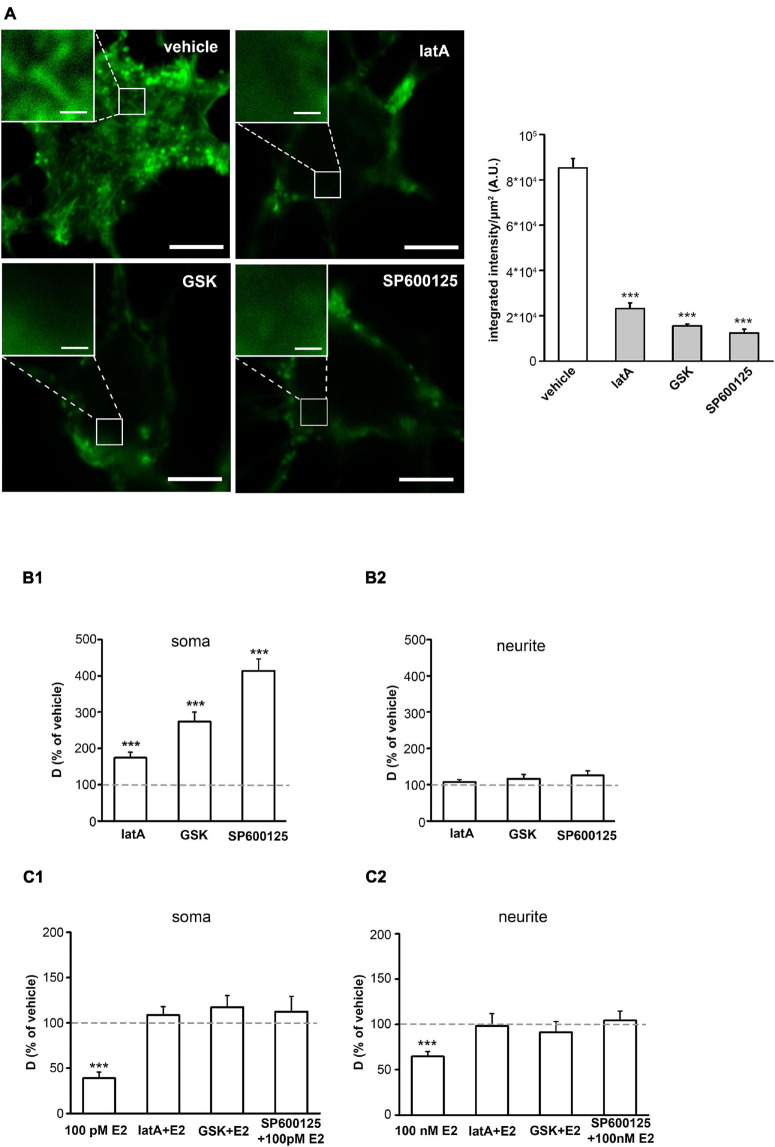
The role of the cortical actin in the rapid effect of E2. **(A)** Left, confocal images depict Alexa Fluor 488 phalloidin-labeled cortical actin network in dPC12 after treatment with vehicle, 1 μM of latA, 1 μM of SP600125 or 1 μM of GSK429286. Scale bar = 5 μm; insert Scale bar = 0.5 μm. Right, the bar graph shows the effect of LatA, GSK429286, and SP600125 on the integrated density of the fluorescently labeled cortical actin network [*n* = 3 cells per group (3 ROIs per cell)]. **(B1,B2)** Effect of LatA, GSK429286, and SP600125 treatment on D_AMPAR_ (% of vehicle treatment as the mean ± SEM; *n* = 215–544 trajectories). **(C1,C2)** Effect of 100 pM of E2 on somas and 100 nM of E2 on neurites with or without LatA, GSK429286, and SP600125 (% of vehicle treatment as the mean ± SEM; *n* = 184–277 trajectories). ***p < 0.001.

First, we validated whether latA, or ROCK and JNK inhibitors altered the morphology of cortical actin. Phalloidin immunostaining demonstrated cortical F-actin in dPC12 (Figure 5A). The density of the cortical actin network in dPC12 was decreased by latA, GSK429286, or SP600125 administration (Figure 5A). In single-molecule tracking experiments, 10 min of latA, or pretreatment with GSK429286 or SP600125 for 60 min significantly increased D_AMPAR_ on soma (vehicle D_AMPAR_ mean ± SEM [μm^2^/s]: 0.021 ± 0.002, Figure 5B1) without affecting D_AMPAR_ on neurites in dPC12 (vehicle D_AMPAR_ mean ± SEM [μm^2^/s]: 0.049 ± 0.003, Figure 5B2). Pretreatment with latA, GSK429286, or SP600125 decreased the effect of 100 pM of E2 on soma and 100 nM of E2 on neurites on the surface movement of GluR2-AMPAR molecules (D_AMPAR_ mean ± SEM [μm^2^/s] on soma: vehicle E2: 0.03 ± 0.004; vehicle E2+latA: 0.062 ± 0.006; vehicle E2+GSK429286: 0.087 ± 0.007; vehicle E2+SP600125: 0.093 ± 0.008; on neurites: vehicle E2: 0.074 ± 0.006; vehicle E2+latA: 0.06 ± 0.004; vehicle E2+GSK429286: 0.113 ± 0.015; vehicle E2+SP600125: 0.128 ± 0.012, Figures 5C1,C2). In experiments with latA, ROCK, and JNK cRPMI containing 0.1 % DMSO was used as vehicle control. Cell viability was not altered by DMSO nor latA treatment (Supplementary Figure 3).

### E2 Rapidly Decreases the Surface Movement and Increases the Synaptic Dwell Time of GluR2-AMPAR in Mouse Primary Hippocampal Neurons

**FIGURE 6:**
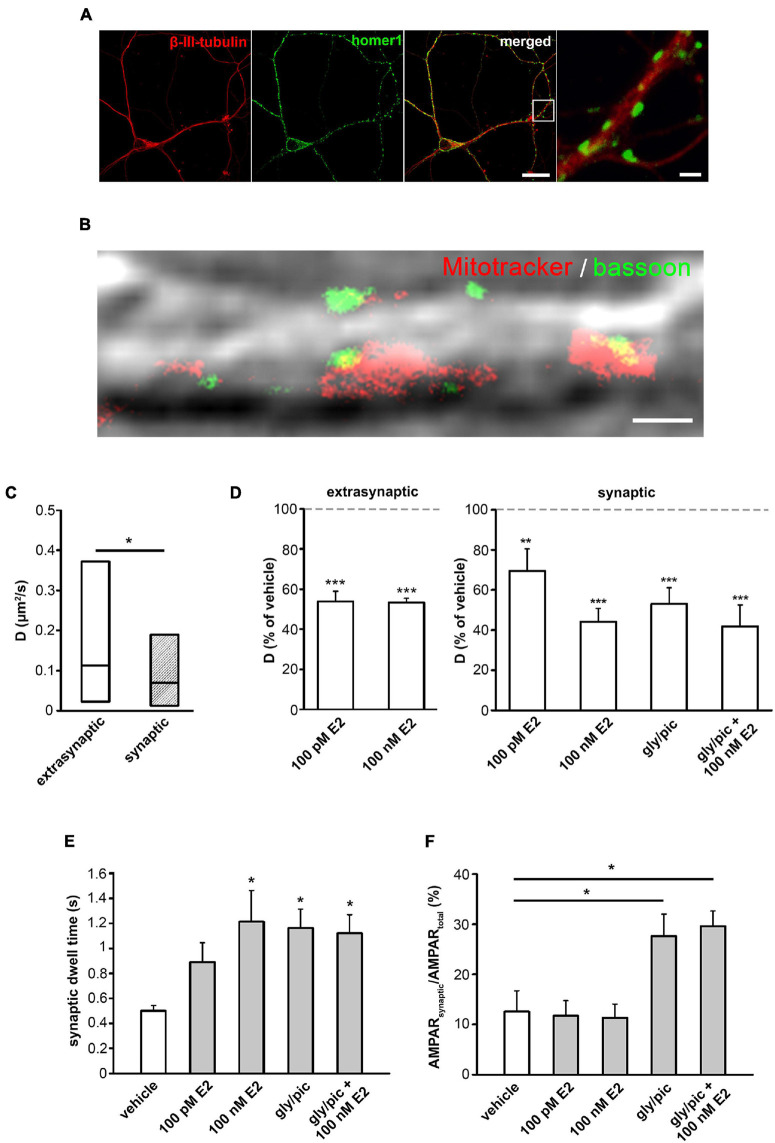
Effect of E2 on the surface movement of GluR2-AMPA on primary hippocampal neurons. **(A)** Photomicrograph shows a primary hippocampal neuron labeled with homer-1 (synapse) and β-III tubulin (neuron). Scale bar = 10 μm, insert Scale bar = 2 μm. **(B)** Dual color STED image of a hippocampal neuron overlayed to differential interference contrast microscopy image depicts live-cell synapse labeling MitoTracker Deep Red (red) and presynaptic protein bassoon (green). Scale bar = 1 μm. **(C)** Distribution of D values of extrasynaptic and synaptic GluR2-AMPAR under control conditions (median ± IQR, *n* = 754 extrasynaptic trajectories and *n* = 104 synaptic trajectories). **(D)** Effect of E2 (100 pM and 100 nM) on D of extrasynaptic and synaptic GluR2-AMPA with or without chemical LTP (cLTP) induced by glycine/picrotoxin (gly/pic) (% of vehicle treatment as the mean ± SEM; *n* = 742–928 extrasynaptic trajectories and *n* = 104–155 synaptic trajectories). **(E,F)** Effect of vehicle, E2 (100 n, 100 pM) with or without cLTP (gly/pic) on synaptic dwell time (mean ± SEM (s); *n* = 104–155) **(E)** and relative surface distribution of synaptic GluR2-AMPAR content (synaptic/total GluR2-AMPA molecule trajectories) (mean ± SEM, *n* = 8–18 recordings) **(F)**. *p < 0.05; **p < 0.01; ***p < 0.001.

To validate the effect of E2 on the surface movement of GluR2-AMPAR in another *in vitro* neuron system and examine the effect of E2 on synaptic GluR2-AMPAR, we performed single-molecule tracking experiments on primary hippocampal neuron culture (Figure 6A).

Immunocytochemical labeling revealed that β-III tubulin-expressing hippocampal neurons have multiple homer-1 positive synapses along their neurites at day *in vitro* 18–21 (Figure 6A). The live-cell presynaptic MitoTracker Deep Red labeling was validated with co-immunostaining of presynaptic protein bassoon. STED imaging showed that every single MitoTracker Deep Red labeled synapse exhibited colocalization with presynaptic marker bassoon. Only 10% of the bassoon labeled synapses showed no colocalization with MitoTracker Deep Red labeling (Figure 6B).

Our single-molecule imaging experiment revealed the surface movement of ATTO 488-labeled GluR2-AMPAR on neurites in extrasynaptic (Supplementary Movie 6) and synaptic (Supplementary Movie 7) regions. D values of GluR2-AMPAR molecules were significantly lower in synapse compared to extrasynaptic regions (Figure 6C). Fluorescence intensity histograms and step sizes for photobleaching suggest that most of the spots represented single fluorophores and single receptors (Supplementary Figure 4). Our *in vivo* labeling failed to show GluR2-AMPAR molecules on soma of hippocampal neurons using highly illuminated laminated optical sheet microscopy (HILO; data not shown).

Both 100 pM and 100 nM of E2 decreased extrasynaptic and synaptic D_AMPAR_ in neurites (Figure 6D). Similar to E2, chemical strengthening of synapses [chemical long term potentiation (cLTP)] elicited a decrease in synaptic D_AMPAR_ (Figure 6D) (vehicle D_AMPAR_ mean ± SEM (μm^2^/s): synaptic: 0.253 ± 0.038, extrasynaptic: 0.247 ± 0.014). Furthermore, 100 nM, but not 100 pM of E2, increased the synaptic dwell time of GluR2-AMPAR to a similar extent as cLTP (Figure 6D). Treatment with 100 nM of E2 did not change the cLTP-induced increase in the synaptic dwell time of GluR2-AMPAR. E2 (100 nM, 100 pM) did not affect synaptic AMPAR content (Figure 6E), and it did not alter cLTP-induced increase in synaptic AMPAR content (Figure 6F).

## Discussion

We found that E2 rapidly decreased the D_AMPAR_ in live dPC12 via rapid membrane-initiated GPER1 signaling in neurites but both GPER1 and ERβ was required for the effect of E2 in soma. Nevertheless, different dose was effective on soma compared to neurites. On soma 100 pM E2 while on neurites 1 nM or 100 nM E2 decreased the D_AMPAR_. This difference may be the consequence of GPER1 internalization in soma induced by 100 nM E2. We show that D_AMPAR_ is affected by the cortical actin network in dPC12 cells. Furthermore, the effects of E2 on D_AMPAR_ in soma and neurites were mediated by actin via the ROCK-cofilin and JNK-cofilin pathways. Importantly, we confirmed our results on dPC12 showing that E2 also decreases D_AMPAR_ in live hippocampal neurons. Similarly, to cLTP induction, E2 decreases D_AMPAR_ and increases the synaptic dwell time of GluR2-AMPAR.

PC12 cells offer an extensively used model in neurobiology as they exhibit some features of mature dopaminergic neurons and in the presence of NGF they differentiate into sympathetic ganglion neurons (dPC12) morphologically and functionally (Wiatrak et al., 2020). Previous experiments demonstrated that dPC12 cells have action potential (Hu et al., 2018), and express GluR2-AMPA, mGluR1 mRNA and protein (Kane et al., 1998; Mehmood et al., 2013). Our results confirmed that dPC12 has abortive action potential similar to immature neurons with moderate amount sodium current (Belinsky et al., 2011) and expresses GluR2-AMPAR and mGluR1 in soma and neurites, providing an effective platform to examine the surface movement of glutamate receptors. Our single-molecule tracking experiments showed that glutamate receptors exhibit either Brownian or confined motions on dPC12 cells. The functional consequence of a changing the diffusion mode is receptor type dependent. For instance, tyrosine receptor kinase A has been shown to induces signaling during immobile phase (Shibata et al., 2006). However, AMPARs become confined when they are trapped inside the synapse in order to strengthen its efficiency (Ehlers et al., 2007). Although previous findings demonstrated that dPC12 exhibits synapse-like structures (Jeon et al., 2010), it does not form classical synapses. Therefore, we used cultured hippocampal neurons to study synaptic GluR2-AMPAR. Our results demonstrated that these neurons were effectively labeled with pre- and postsynaptic markers, MitoTracker Deep Red and homer-1, respectively. Experiments performed by Ehlers et al. (2007) demonstrated that *in vivo* MitoTracker labeling exhibited around 84% colocalization with the presynaptic marker bassoon. Our immunofluorescence stainings showed that MitoTracker Deep Red entirely colocalized with bassoon, although some synapses were labeled with bassoon alone in our hippocampal culture. In agreement with previous studies (Groc et al., 2008) our results demonstrated that synaptic D_AMPAR_ is lower than extrasynaptic D_AMPAR_ suggesting that GluR2-AMPAR exhibited a more confined motion in the synapses.

### Compartment Specific E2 Action on the Surface Movements of GluR2-AMPAR

Besides its classical genomic action, E2 exerts rapid non-classical effects on glutamate receptors. The surface movement of glutamate receptors plays critical roles in functions, such as glutamatergic neurotransmission and synaptic plasticity. It has been described that AMPAR, the most abundant glutamate receptor in excitatory synapses, showed immobile or relatively slow diffusion in the postsynaptic density but exhibited Brownian movement outside the synapse (Borgdorff and Choquet, 2002). It was also reported that E2 decreased the surface movement of GluN2-N-methyl-D-aspartate receptors (NMDA) (Potier et al., 2016). However, the effect of E2 on surface movement of AMPAR is unknown. In this study, we examined whether E2 alters the surface movement of GluR2-AMPAR molecules, the most ample AMPAR subunit in neurons. Here, we show that E2 decreases D_AMPAR_ in a concentration-dependent manner, with distinct effects on soma and neurites in dPC12. However, E2 altered only D_AMPAR_ but not D_mGluR1_, suggesting that the rapid modulation of glutamatergic receptor surface diffusion by E2 is type-dependent. It is worth noting that the rapidity of E2 action on D_AMPAR_ (≤5 min) indicates a non-classical mechanism.

ERs, namely GPER1, ERα, and ERβ, are of great interest and have been suggested to be involved in non-classical E2 actions. Our PCR results showed the expression of GPER1 and ERβ but not ERα in dPC12. Interestingly, our experiments with ER agonists and antagonists demonstrated a compartment-specific effect on dPC12, as they have different effects on soma and neurites. In soma, the ability of E2 to reduce D_AMPAR_ requires both ERβ and GPER1 since this response was observed after the co-application of ERβ and GPER1 agonists (DPN and G1) but not after application of DPN or G1 alone. The complementary effect of liganded ERβ and GPER1 on soma is also corroborated by the fact that GPER1 blocker G15 inhibits the effect of E2 on somatic D_AMPAR_. In contrast, on neurites G1 reduced D_AMPAR_, DPN was not effective, and G15 antagonized the effect of E2. In summary, both ERβ and GPER1 are required for E2 effect on soma, but on neurite E2 effect occurs through GPER1 only. Studies have revealed that cortical actin network differs in soma and neurite and its dynamics is regulated by ERβ (Zhao et al., 2017). As discussed later, we found in dPC12 that actin structure influenced the membrane movement of receptors differently on soma and neurite. We assume that on soma ERβ and GPER1 regulates receptor dynamics through cortical actin rearrangement, while on neurite GPER1 alone affects receptor movements via an unknown mechanism unrelated to cortical actin network.

The concentration dependence of E2 action differs between soma and neurites in dPC12. While 100 pM of E2 reduced D_AMPAR_ in soma, higher concentrations (1 nM or 100 nM) were required to decrease the D_AMPAR_ in neurites. One possible reason for the compartement-specific E2 action may be the differences in the distribution of GPER1 molecules on the membrane. Indeed, our STORM experiments showed that the GPER1/GluR2-AMPAR ratio was higher in soma than in neurites, indicating that neurites express less GPER1 than soma do. These observations are consistent with our finding showing a significant decrease in D_AMPAR_ in neurites after exposure to high E2 (1 nM and 100 nM).

Interestingly, high doses of E2 (1 nM and 100 nM) did not alter D_AMPAR_ in soma. Previous studies have indicated that GPER1 undergoes desensitization after the administration of the ligand at high concentrations (Brailoiu et al., 2007). Thus, it is likely that a high concentration of E2 induces GPER1 desensitization in the soma. Previous experiments demonstrated that E2 administration could induce translocation of GPER1 from the cell membrane to the cytoplasm (*33, 34*), resulting in the desensitization of the receptor (Filardo and Thomas, 2012). Our STED experiments corroborated these findings because 10 min after administration of 100 nM of E2, GPER1 immunolabeling relocated from the membrane region to the cytoplasm (Funakoshi et al., 2006), indicating a rapid internalization of GPER1 on soma. Rapid internalization indicates the desensitization of GPER1, which may explain why high doses of E2 were ineffective on the soma. The lack of GPER1 internalization on neurites may be the consequence of the low expression level of GPER1. We hypothesize that an even higher concentration of E2 would be sufficient to induce internalization due to the low level of GPER1.

### Role of Cortical Actin in the Effect of E2 on the Surface Movement of GluR2-AMPARs

It has been shown earlier that the actin cytoskeleton can interact with the intracellular domains of membrane receptors, thus regulating their movement (Kusumi et al., 2014). Single-particle tracking studies of lipid-anchored molecules demonstrated reduced mobility in the axon initial segment and that the confined motion was due to actin structures (Albrecht et al., 2016). Our present findings confirm these previous observations (Hanley, 2014), as the disruption of cortical actin by latA increased D_AMPAR_ in soma. Interestingly, latA has a compartment-specific effect because it is not effective in neurites. Furthermore, we found that D_AMPAR_ and D_mGluR1_ were higher for neurites than for soma. Super-resolution imaging studies revealed that soma and neurites have different cortical actin structures (Lukinavičius et al., 2014; Han et al., 2017). Actin has a polygonal lattice structure in soma (Han et al., 2017), and its associated proteins such as adducin and spectrin form 190-nm-spaced ring-like structures around the circumference of neurites (Xu et al., 2013; Han et al., 2017). We hypothesize that the higher D values measured on neurites arise from the difference between the structural arrangement of actin in soma and neurites. This may also provide an effective basis for the compartment-specific effect of latA and surface dynamics of GluR2-AMPARs.

Recent evidence implicates that cortical actin is important in receptor crosstalk through modulation of protein dynamics (Mattila et al., 2016). Cofilin is a highly abundant constitutively active actin-binding protein that alters the properties of F-actin and is regulated by the ROCK-cofilin and JNK-cofilin pathways (Hu et al., 2018; Kim et al., 2019). Phosphorylation inactivates cofilin and facilitates actin filament assembly. E2 increases the activity of cofilin (Kramár et al., 2009a; Brandt and Rune, 2019) and stabilizes the F-actin cytoskeleton via GPER1 (Wang et al., 2019). Cofilin has been reported to mediate cortical actin dynamics that regulate AMPAR trafficking in synaptic plasticity (Gu et al., 2010). Therefore, we investigated the role of actin in the effect of E2 on D_AMPAR_. Our results demonstrated that latA diminished the effect of E2, indicating that cortical actin plays a pivotal role in E2 action on D_AMPAR_. Our results also demonstrated that the E2-induced decrease in D_AMPAR_ is completely blocked by the inhibition of the ROCK-cofilin or JNK-cofilin pathways in soma and neurites. We suggest that E2 binding to GPER1 activates both the ROCK-cofilin and JNK-cofilin pathways, which then change the cortical actin dynamics and decrease the surface movement of GluR2-AMPAR.

### Effect of E2 on D_AMPAR_ in the Hippocampal Neurons

The pressing question related to the rapid E2 effect on AMPARs is that of explaining the physiological relevance of the observed changes.

To confirm the effect of E2 on D_AMPAR_ in another *in vitro* neuron system and examine the effect of E2 on synaptic GluR2-AMPAR, we performed single-molecule tracking experiments on a primary hippocampal neuron culture. Cultured hippocampal neurons expressing ERα, ERβ, and GPER1 (Wehrenberg et al., 2001; Prange-Kiel et al., 2003; Zhao et al., 2016) provide physiologically relevant *in vitro* model for studying E2 effect. Our results showed that E2 administration (100 pM and 100 nM) rapidly decreased the synaptic and extrasynaptic D_AMPAR_ in hippocampal neurons similar to dPC12.

Long term potentiation of excitatory synaptic transmission is a well-known form of synaptic plasticity and is considered a cellular model for learning and memory. Although several studies have demonstrated that E2 plays an essential role in LTP and alters memory formation (Spencer et al., 2008; Fester and Rune, 2015), the precise molecular mechanism is not clear. AMPAR plays a pivotal role in synaptic alterations involved in synaptic transmission, synaptic plasticity, LTP, learning, and memory. Using single-molecule tracking experiments and AMPAR immobilization techniques, Penn et al. (2017) have shown that the surface movement of AMPARs is a key factor in the modulation of synaptic potentiation and learning (Phan et al., 2015). At the molecular level, the recruitment and slow diffusion of glutamate receptors at the postsynaptic site have been shown after LTP (Kovács et al., 2018). Indeed, our single-molecule tracking of hippocampal neurons demonstrated that cLTP decreased D_AMPAR_ in synapses and increased the synaptic dwell time and content of GluR2-AMPARs. Similar to cLTP, 100 nM of E2 decreased D_AMPAR_ and increased the dwell time of GluR2-AMPA in the synapse. Although recent morphological studies have demonstrated that E2 increased the expression of GluR2 in mushroom spines at 120 min *in vivo* (Avila et al., 2017) our results show that E2 did not affect the GluR2-AMPAR content in the synapses within 20 min. We suggest that E2 can rapidly enhance the synaptic efficacy of glutamatergic synapses by decreasing D_AMPAR_. Interestingly, E2 did not change the effect of cLTP on D_AMPAR_, dwell time, and synaptic content of GluR2-AMPAR. However, E2 can likely increase the efficacy of cLTP by retaining the AMPARs in the synapses.

## Conclusion

Our study demonstrates that E2 rapidly and dose-dependently decreases the surface movement of GluR2-AMPARs via compartment-specific ER-mediated mechanisms in live neurons. Our results also suggest that cortical actin mediates liganded GPER1 action on the surface movement of GluR2-AMPARs via the ROCK-cofilin and JNK-cofilin pathways. This study provides the first evidence that E2 decreases the surface movement and increases the dwell time of GluR2-AMPARs in the synapses. These results provide a strong foundation for understanding the molecular mechanism by which E2 affects neuronal plasticity and glutamatergic neurotransmission. Finally, these observations will likely be of physiological importance for cognitive functions and of particular relevance to E2 action on memory formation.

## Materials and Methods

### Cell Culture and Neuronal Differentiation

For single-molecule tracking of glutamate receptors, rat pheochromocytoma cells (PC12, Sigma-Aldrich) were differentiated into dPC12. PC12 cells were plated at a density of 2 x 10^3^ cells/cm^2^ on collagen IV-coated 35-mm glass-bottom dishes (MatTek Corporation, Ashland, MA, United States) in phenol red-free RPMI 1640 medium supplemented with 10% horse serum (HS), 5% fetal bovine serum (FBS), and 2 mM L-glutamine (culture RPMI, cRPMI). Twelve hours after plating, the medium was replaced with phenol red-free RPMI 1640 medium supplemented with 1% HS, 2 mM L-glutamine, and 50 ng/mL nerve growth factor (NGF-2.5S, Sigma-Aldrich, St. Louis, MO, United States). The cells were fed with dRPMI after 2 days and used for imaging after 4 days of differentiation.

For antibody specificity testing chinese hamster ovary cells (CHO) were cultured in phenol-red free F12 medium supplemented with 10% fetal bovine serum (FBS), and 2 mM L-glutamine (culture F12, cF12). A day before transfection 2 x 10^5^ CHO cells were plated onto untreated coverslip.

Cultures of the hippocampal neurons were prepared from C57BL/6 mouse embryos (E17-18) to examine the surface movement of extrasynaptic and synaptic GluR2-AMPAR molecules. The brains were aseptically removed from the skull, meninges were pulled off, and both hippocampi were separated from the cortex. Dissected hippocampi were incubated in pre-warmed MEM (Thermo Fisher Scientific) containing 0.05% trypsin (Gibco) and 0.05% DNaseI (Gibco) at 37°C for 15 min. Two milliliters of FBS was added to stop the digestion, and the mixture was centrifuged for 5 min at 1200 rpm. Cells were triturated in Neurobasal (NB, Thermo Fisher Scientific) supplemented with B27 (Thermo Fisher Scientific), 5% FBS, 1% penicillin-streptomycin (Thermo Fisher Scientific). Then, 100.000 cells were plated on glass bottoms coated with poly-D-lysine (PDL)- and laminin-coated 35-mm glass-bottom dishes (Kovács et al., 2018). Neurons were cultured in an incubator at 95% relative humidity and 5% CO_2_. After 3 days of seeding, one-third of the medium was replaced with pre-warmed MEM every third-day until day *in vitro* 19–21.

### Validation of the Neuronal Differentiation of PC12 and Synapses on Hippocampal Neurons

To validate the neuronal differentiation of PC12 cells, immunofluorescent staining was performed with microtubule-associated protein 2 (MAP2) and β-III tubulin antibodies. Cells were fixed in 4% paraformaldehyde (PFA) for 15 min and permeabilized with 0.03% Triton X-100 for 30 min after 4 days of differentiation. The cells were then incubated overnight at 4°C with either mouse anti-MAP2 antibody (1:1000, MAB3418, Millipore) or mouse neuron-specific anti-β-III tubulin antibody (1:1000, MAB1195, RD Systems), before being incubated with biotinylated donkey anti-mouse F(ab’)_2_ (1:200, Jackson ImmunoResearch) and Alexa Fluor 647-conjugated streptavidin (1:2000, Thermo Fisher Scientific).

The electrophysiological properties of dPC12 were tested using whole-cell patch-clamp recording. Patch pipettes (1.5 mm outer diameter and 1.1 inner diameter) with a resistance of 6 MΩ were pulled from borosilicate glass capillaries with a micropipette puller (Sutter Instruments). The pipette recording solution contained (in mM) 10 KCl, 130 K-gluconate, 1.8 NaCl, 0.2 EGTA, 10 HEPES, and 2 Na-ATP, 0.2% biocytin and the pH was adjusted to 7.3 with KOH. All recordings were performed at 32°C in a chamber perfused with oxygenated artificial cerebrospinal fluid (ACSF) containing (in mM) 2.5 KCl, 10 glucose, 126 NaCl, 1.25 NaH_2_PO_4_, 2 MgCl_2_, 2 CaCl_2_, and 26 NaHCO_3_. Whole-cell recordings were made with an Axopatch 700B amplifier (Molecular Devices) using an upright microscope (Nikon Eclipse FN1) equipped with infrared differential interference contrast optics. Cells with access resistance below 20 MΩ were used for analysis. Signals were low-pass filtered at 5 kHz and digitized at 20 kHz (Digidata 1550B, Molecular Devices). Acquisition and subsequent analysis of the data were performed using Clampex9 and Clampfit software (Axon Instruments). After measurement cells were fixed with 4% PFA for 15 min and permeabilized with 0.03% Triton X-100 for 30 min and Alexa Fluor 488 conjugated Streptavidin (1:2000) was applied for 2 h at room temperature.

Dual-label immunofluorescence was performed to detect mature synapses in hippocampal neurons (Figure 6A). Cells were treated as described above except that they were incubated overnight at 4°C with anti-homer1 (1:1000, 160006, Synaptic Systems) and anti-β-III tubulin (1:1000, MAB1195, RD Systems) antibodies followed by Alexa Fluor 488-conjugated anti-chicken antibody and Alexa Fluor 647-conjugated anti-mouse antibody, respectively.

All immunofluorescence images were taken on CLSM (Zeiss LSM710, 100X). A helium-neon laser with 488 and 633 nm wavelength was used to excite Alexa Fluor 488 and Alexa Fluor 647, respectively. Images were captured at 2048x2048 pixel resolution with a 2 μm optical thickness.

We applied MitoTracker Deep Red, carbocyanine-based MitoTracker dye, for synaptic labeling of live neurons. Previous experiments showed that MitoTracker effectively labels mitocondria live presynaptic terminals (Ehlers et al., 2007). To validate Mitotracker Deep Red as a synapse labeling in our experiments, hippocampal neurons were incubated with MitoTracker Deep Red (1 nM, Thermo Fisher Scientific) at 37°C for 10 min. After washing neurons were fixed as described above and incubated overnight at 4°C with anti-bassoon antibody (1:1000, ab82958, Abcam) followed by abberior STAR ORANGE conjugated anti-mouse secondary antibody (1:500, STORANGE, Abberior). 2 dimensional stimulated emission depletion (STED) images were taken on Abberior Expert Line STED system equipped with Plan Apo 100X/1.45 objective (Nikon). STAR ORANGE and MitoTracker were excited at 561 nm and 640 nm, respectively. The wavelength of the depletion laser was 775 nm. Super-resolution images were captured with 20 nm pixel size, 20 ms dwell time, and the pinhole was set to 1 A.U.

### Detection of Estrogen Receptors

Expression levels of estrogen receptor α (ERα), estrogen receptor β (ERβ), and the membrane estrogen receptor, GPER1, were examined in the dPC12. Total ribonucleic acid (RNA) was extracted from dPC12 with a conventional TRIzol (Thermo Fisher Scientific)-based protocol, and complementary deoxyribonucleic acid (cDNA) was constructed using a High-Capacity RNA-to-cDNA Kit (Thermo Fisher Scientific). The following polymerase chain reaction (PCR) primers were used: ERα, 5′-CGTAGCCAGCAACATGTCAA-3′, and 5′-AATGGGCACTTCAGGAGACA-3′; ERβ, 5′-GAGGTGC TAATGGTGGGACT-3′ and 5′-CTGAGCAGATGTTCCAT GCC-3′; and GPER1, 5′-TGCACCTTCATGTCCCTCTT-3′ and 5′-AAGGACCACTGCGAAGATCA-3′.

### Glutamate Receptor Labeling in Live dPC12 and Primary Hippocampal Neurons

To detect GluR2-AMPAR and mGluR1 molecules in the plasma membranes of dPC12, live-cell immunofluorescent labeling was performed. Before single-molecule imaging, dPC12 were incubated in dRPMI with ATTO 488-labeled antibodies directed against the extracellular N-terminal domain of either rat GluR2 (1:100, Alomone Labs) or rat mGluR1 (1:100, Alomone Labs) at 37°C for 6 min. Specificity of ATTO 488-labeled GluR2-AMPAR antibody has been reported previously in brain sections of GluR2 knockout mice (Egbenya et al., 2018). The specificity of the antibodies was also tested with control peptides (GluA2_179–193_ peptide and mGluR1_501–516_ peptide, Alomone Labs), and no immunoreactivity was observed (Supplementary Figure 5). In order to further test the specificity of anti-GluR2 antibody CHO cells were transfected with plasmid encoding GluR2 subunit using Lipofectamine 3000 (Sigma) according to the manufacturer’s protocol. Rat GluR2 cDNA sequence was subcloned into a pCl mammalian expression vector under XhoI-NotI place. The GluR2 cDNA sequence was a gift from Jeremy Henley (Addgene plasmid #64941). The construct was verified with Sanger sequencing. 24 h after transfection cells were labeled and imaged the same manner as detailed above. Supplementary Movie 8 shows the movements of ATTO 488-labeled GluR2 subunits in the membrane of a transfected CHO cell. The omission of GluR2 subunit transfection resulted in complete absence of ATTO 488 labeling (Supplementary Movie 8).

To simultaneously label live synapses and GluR2-AMPAR, cultured hippocampal neurons were incubated in MEM containing MitoTracker Deep Red (1 nM, Thermo Fisher Scientific) and ATTO 488-labeled antibodies directed against the extracellular N-terminal domain of rat GluR2 (1:100, Alomone Labs) at 37°C for 10 min. Neurons were imaged after they were carefully washed 3 times with pre-warmed MEM.

### Drug Application and Cell Viability Detection

The following drugs were applied immediately before imaging the dPC12 in dRPMI: 17β-estradiol (E2, Sigma-Aldrich, 100 pM in 10^–5^% EtOH, 1 nM and 100 nM in 10^–3^% EtOH); G1, a selective GPER1 agonist [Tocris, 100 nM in 10^–5^% dimethyl sulfoxide (DMSO)] (Sárvári et al., 2009); and diarylpropionitrile (DPN), a selective ERβ agonist (Tocris, 10 pM in 2 x 10^–5^% DMSO) (Bálint et al., 2016). To block GPER1, dPC12 were incubated in dRPMI containing G15, a selective GPER1 antagonist (Tocris, 1 μM in 2x10^–3^ % DMSO) (Sárvári et al., 2009), for 10 min before E2 application and imaging. To inhibit actin polymerization, we applied latrunculin A (latA, Sigma-Aldrich, 1 μM in 0.1% DMSO) for 5 min before E2 addition and imaging. We also inhibited the actin polymerization regulator cofilin (Bamburg and Bernstein, 2010), via application of a selective Rho-associated protein kinase (ROCK) inhibitor, GSK429286 (Tocris, 1 μM in 0.1% DMSO) for 1 h (Liu et al., 2018) or selective c-Jun N-terminal kinase (JNK) inhibitor, SP600125 (Tocris, 1 μM in 0.1% DMSO) for 1 h (Kim et al., 2019).

After latA treatment, that is, at the end of the experiments, the viability of the dPC12 was tested with a LIVE/DEAD viability/cytotoxicity kit (Thermo Fisher Scientific) according to the manufacturer’s instructions. The results demonstrated that the cells retained their plasma membrane integrity until the end of the experiments.

The hippocampal neurons were treated with E2 in the same manner as detailed above, with the exception that chemical long term potentiation (cLTP) was induced by incubating the neurons in MEM containing glycine (200 μM) and picrotoxin (1 μM) for 3 min (Groc et al., 2008) at room temperature. After washing 3 times, the cells were placed back at 37°C for 20 min.

### Single-Molecule Imaging of Glutamate Receptors Using Total Internal Reflection Fluorescence and Highly Illuminated Laminated Optical Sheet Microscopy

Single-molecule imaging of labeled glutamate receptors was carried out on an Olympus IX81 fiber TIRF microscope equipped with Z-drift compensation (ZDC2) stage control, a plan apochromat objective (100X, NA 1.49, Olympus), and a humidified chamber heated to 37°C and containing 5% CO_2_.

The dish containing dPC12 was mounted in the humidified chamber of the TIRF microscope immediately after *in vivo* labeling. A 491 nm diode laser (Olympus) was used to excite ATTO 488, and emission was detected above the 510 nm emission wavelength range. The angle of the excitation laser beam was set to reach a 100 nm penetration depth of the evanescent wave.

Hippocampal neurons were imaged using an Olympus IX81 fiber TIRF microscope with HILO illumination (Tokunaga et al., 2008). The ATTO 488 dye was excited with the same laser as described above, and emission was detected with a 518QM32 filter. MitoTracker was excited with a 633 diode laser (Olympus), and emission was detected with a 655WB20 filter. A Hamamatsu 9100-13 electron-multiplying charge-coupled device (EMCCD) camera and Olympus Excellence Pro imaging software were used for image acquisition by TIRF and HILO microscopy.

Experiments were performed for 20 min. During the measurement period of ATTO 488-GluR2-AMPAR and ATTO 488-mGluR1, 20–30 images were recorded with 10-s sampling intervals and 33-ms acquisition times. Single-molecule tracking of ATTO 488-GluR2-AMPAR and ATTO 488-mGluR1 was performed with custom-made software written in C++ (WinATR (Kusumi Lab, Membrane Cooperativity Unit, OIST). The center of each particle was localized by two-dimensional Gaussian fitting, and the trajectory for each signal was created by a minimum step size linking algorithm that connected the localized dots in subsequent images. The trajectories were individually checked, and artifacts or tracks shorter than 15 frames were excluded from further analysis. A minimum of 400 trajectories was collected in each experiment from both the soma and neurites. To examine the effect of E2 or vehicle (EtOH), 100–150 trajectories were collected in every consecutive 5-min interval for up to 20 min (0–5, 5–10, 10–15, and 1–20 min). To identify the live synapses in hippocampal neurons, the MitoTracker Deep Red signal was detected as time-lapse stacks for 10 s. Time-lapse stacks were defined as Z-stacks, and an average intensity Z-projection was applied to increase the image quality and optimize the signal-to-noise ratio of the MitoTracker Deep Red signal.

### Calculation of the Surface Movement Parameters of the Glutamate Receptors

The mean square displacement curve for each trajectory was calculated by the following equation:


$$MSD\left(m\Delta T\right)=\frac{1}{N-m}\sum_{i=1}^{N-m}\left({\left(x_{i+m}-x_{i}\right)}^{2}+{\left(y_{i+m}-y_{i}\right)}^{2}\right)$$

where, *x*_*i*_ and *y*_*i*_ are the coordinates of the signal’s center, Δ*T is* the time interval between two consecutive frames, *N is* the total number of frames, and *m* represents the time delay.

The maximum likelihood estimation (Berglund, 2010) was applied to obtain the corresponding diffusion coefficient (D) value for each trajectory. Δ*x*_k_ and Δ*y*_k_ represent the observed displacements (Δ*x*_*k*_ = *x*_*k* + 1_−*x*_*k*_ and Δ*y*_*k*_ = *y*_*k* + 1_−*y*_*k*_) arranged in *N*-component column vectors, where the total number of frames is equal to *N*+1, and *x*_*n*_ and *y*_*n*_ are the coordinates of the signal center on the *n*th frame. Σ is the *N* × *N* covariance matrix defined by the following equation:


$$\sum_{i\,j}=\left\{ \begin{array}{ccc} 2\,D\,\Delta \,t-2\,\left(2\,D\,R\,\Delta \,t-\sigma^{2}\right),i\,f\,i=j & & \\ 2\,D\,R\,\Delta \,t-\sigma^{2},i\,f\,i=j\pm 1 & & \\ 0,\mathrm{otherwise} & & \end{array}\right.$$

where, D is the diffusion coefficient, Δ*t* is the frame integration time, *σ* is the static localization noise, and *R* summarizes the motion blur effect. In our case, *R* = 1/6 as a consequence of continuous illumination.

The likelihood was defined by the following function:


$$L\left(\Delta x,\Delta y\right)=-log\left|\Sigma \right|-\frac{1}{2}{\left(\Delta x\right)}^{T}\Sigma^{-1}\left(\Delta x\right)-\frac{1}{2}{\left(\Delta y\right)}^{T}\Sigma^{-1}\left(\Delta y\right)$$

D and σ, which provide the maximal likelihood, are the estimated diffusion coefficient and static localization noise, respectively. The calculation of the determinant and the inverse of the covariance matrix at each step of the optimization method can be a severe computational difficulty at high values of *N*. An approximation (Gray, 2006) based on the theory of circulant matrices is applicable (Berglund, 2010). The global optimization of the likelihood function based on this approximation was implemented in MATLAB. The goodness of optimization was judged by evaluating the static localization noise. An optimization was considered to be inaccurate, and the corresponding trajectory was excluded from further analysis when the estimated static localization noise was out of ±90% range of the group’s mean.

To examine the synaptic movements of GluR2-AMPAR, the maximum intensity Z-projected MitoTracker labeled synaptic area was determined manually. GluR2-AMPAR molecules were identified as synaptic if the trajectory was colocalized at least on one frame with the MitoTracker signal, and extrasynaptic if there was no co-localization (Groc et al., 2006, 2007). D values were calculated as described above for both synaptic and extrasynaptic GluR2-AMPAR (Groc et al., 2008). The synaptic dwell time for each treatment was determined as the mean time spent by synaptic receptors within the synaptic (MitoTracker labeled) area. The relative surface distribution of synaptic GluR2-AMPAR content (synaptic/total GluR2-AMPAR molecule trajectories) was calculated for each recording after vehicle or E2 treatment.

### Co-Localization Analysis of GluR2-AMPAR and GPER1 Using Stochastic Optical Reconstruction Microscopy

Super-resolution 3D STORM imaging was performed to examine the number of receptors and the probability of interaction between GluR2-AMPAR and GPER1 in dPC12. PC12 cells were plated onto poly-D-lysine (PDL)- and laminin-coated coverslips (Kovács et al., 2018), and differentiated into neurons as described above. The neurons were incubated in dRPMI medium containing either vehicle (EtOH) or E2 (100 pM or 100 nM) at 37°C for 10 min. Immediately after treatment, GluR2-AMPAR was applied to live PC12 cells with mouse anti-GluR2-AMPAR antibody (1:1000, MAB397, raised in mouse, Millipore) at 37°C for 20 min, followed by fixation in 4% paraformaldehyde (PFA). After a thorough wash, the cells were incubated with anti-GPER1 primary antibody (1:5000, AF5534, Novus Biological) at 4°C for 48 h. CF-568-labeled donkey anti-goat antibody (1:400, Biotium) was applied at room temperature for 2 h. Following three consecutive washes, Alexa Fluor 647-labeled anti-mouse antibody was applied at room temperature for 2 h (1:200, Jackson ImmunoResearch). The coverslips were washed, covered with imaging medium prepared from the following reagents in Dulbecco’ PBS: 5% glucose, 0.1 M mercaptoethylamine, 1 mg/mL glucose oxidase and μl/mL 2.5 catalase (1500 U/mL) (Dudok et al., 2015), and transferred onto standard glass slides immediately before imaging. Using a CFI Apochromat TIRF 100X objective, corresponding confocal and super-resolution images were collected with a Nikon N-STORM/C2+ super-resolution system based on the platform of a Nikon Ti-E inverted microscope equipped with Nikon Perfect Focus System and a Nikon C2 confocal scan head. 3D STORM images were captured with an Andor iXon Ultra 897 EMCCD camera (pixel size: 160 nm/pixel) using an astigmatic imaging method which enables us to localize molecules within an axial distance of −300 to 300 nm from the center plane. STORM images were acquired by illuminating the samples with high-power lasers (561 and 647 nm). Image acquisition and processing were performed using the Nikon NIS-Elements AR software with the N-STORM module. The obtained 3D STORM localization points were filtered for the collected photon number, z-position (within an axial distance of −300 to 300 nm from the center plane), and local density using the VividSTORM software (Barna et al., 2016). Localization points were selected according to the regions of interest (ROIs) that were manually defined based on the correlated high-resolution confocal images. The clusters of selected localization points were determined using the density-based spatial clustering of applications with noise (DBSCAN) algorithm. A cluster was defined if 3 or more localization points were detected within a 100 nm radius. The center of mass representing a single molecule was calculated for each cluster. In order to examine the number of GPER1 molecules relative to GluR2-AMPAR molecules, the ratio between the number of GPER1 and GluR2-AMPAR molecules (GPER1/GluR2-AMPAR) was calculated for both the soma and neurites.

### Analysis of the Subcellular Distribution of GPER1 in dPC12 Using 2D-STED Microscopy

To examine whether GPER1 is internalized after E2 administration, super-resolution 2D-STED microscopy was used. After 10 min of treatment with vehicle (10^–3^% EtOH) or 100 nM, E2 dPC12 was fixed with 4% PFA. Then, GPER1 immunocytochemistry was performed in the same manner as detailed in the section on STORM, with the exception that Alexa Fluor 647 conjugated anti-goat secondary antibody was used (1:2000) to visualize GPER1. To determine the boundary between the membrane and cytoplasm, dPC12 were treated with a vehicle or 100 nM E2 and cell surface biotin labeling was performed prior to GPER1 immunocytochemistry. Cells were washed with PBS containing 1 mM Ca^+^ and 1 mM Mg^+^ and incubated with biotin (0.5 mg/mL in PBS, EZ-Link Sulfo-NHS-LC-Biotin, Thermo Fisher Scientific) for 10 min at room temperature followed by wash and fixation with 4% PFA for 20 min. After washing, cells were incubated with Alexa Fluor 594 conjugated streptavidin (1:2000, Thermo Fisher Scientific) for 20 min at room temperature. STED images were taken as described above. Based on the result of STED microscopy, 1 μm thick membrane area was defined from the outer edge of GPER1 signal (Figures 4C2,D). For image analysis of GPER1 internalization we used cells labeled with GPER1 antibody alone. The captured images were analyzed using Fiji (Schindelin et al., 2012). After background subtraction, the mean intensity value was calculated with the plot profile algorithm within a specified rectangle (ROI size: 12 μm^2^) (Figures 4C2,E). From each cell (*n* = 15 total) one ROI (with 2 μm^2^ membrane and 10 μm^2^ cytoplasmic area) was selected, integrated density was calculated and normalized to the area (μm^2^) (Figure 4F).

### Imaging of the Cortical Actin Morphology

To validate the effect of latA, GSK429286 and SP600125 on dPC12, the morphology of the cortical actin network of dPC12, were examined after drug administration. After 10 min of treatment with 1 μM of latA, or after 60 min of treatment with 1 μM of GSK429286, 1 μM of SP600125, or vehicle (in 10^–3^% DMSO), dPC12 were fixed in 4% PFA, permeabilized with 0.1% Triton X-100 for 30 min, and incubated with Alexa Fluor 488 phalloidin (1:200, Thermo Fisher Scientific) for 45 min at room temperature. Imaging was performed on CLSM (Zeiss LSM710, 100X), and Alexa Fluor 488 was excited with an argon laser at a wavelength of 488 nm. Images with 2 μm optical thickness and 4096x4096 (X/Y) resolution were captured with the use of ZEN software applying the same settings (laser power, digital gain) to all images. 6 cells were selected from each treatment group (vehicle, latA, GSK, SP6001235). Three ROIs (ROI size: 4.3 μm^2^) were selected from each cell and the average integrated density was calculated from raw images using FIJI software. Results are expressed in the percentage of ROI in order to obtain the integrated density values per μm^2^ (in arbitrary units).

### Statistics

To compare the surface movements of GluR2-AMPAR and mGluR1 in soma and neurites, D values were expressed as cumulative probability functions. In the rest of the experiments, the D values were expressed as the mean percentage of control (vehicle) + SEM in figures. GPER1/AMPAR ratios and extrasynaptic/synaptic D_AMPAR_ values were expressed as the median±25–75% (interquartile range). To compare the distributions of D values of vehicle control and treatment and extrasynaptic/synaptic D_AMPAR_ values the Kolmogorov-Smirnov test was used. The integrated GPER1/AMPAR ratios of the soma and neurites and densities of Alexa Fluor 488-phalloidin and Alexa Fluor 647-GPER1 immunolabeling were compared with the Mann-Whitney U test. Synaptic dwell time and exchange frequency of GluR2-AMPAR were compared using the Kruskal-Wallis test followed by Dunn’s post hoc test. Statistical differences were considered significant at a *p*-value of < 0.05. All statistical analyses were performed with Statistica version 13.3 for Windows (TIBCO Software Inc., CA, United States).

## Data Availability Statement

The original contributions presented in the study are included in the article/Supplementary Material, further inquiries can be directed to the corresponding author.

## Ethics Statement

The animal study was reviewed and approved by Animal Welfare Committee of University of Pécs, Hungary.

## Author Contributions

SG, KB, TK, DE, GK, BO, GM, TJ, MK, CV, and FL performed experiments. SG, KB, TK, DE, GK, BO, GM, TJ, MK, CV, FL, TF, AK, and IÁ developed the methodology. SG, TJ, GM, TF, and AK analyzed the data. IÁ and SG designed the experiments. SG, KB, and IÁ wrote the manuscript. IÁ obtained funding. All authors contributed to the article and approved the submitted version.

## Conflict of Interest

The authors declare that the research was conducted in the absence of any commercial or financial relationships that could be construed as a potential conflict of interest.

## Publisher’s Note

All claims expressed in this article are solely those of the authors and do not necessarily represent those of their affiliated organizations, or those of the publisher, the editors and the reviewers. Any product that may be evaluated in this article, or claim that may be made by its manufacturer, is not guaranteed or endorsed by the publisher.
