# Supplementary material for: Single-Molecule Imaging Reveals Rapid Estradiol Action on the Surface Movement of AMPA Receptors in Live Neurons
Source: Front Cell Dev Biol. 2021 Sep 23;9:708715. doi: 10.3389/fcell.2021.708715 (PMC8495425; doi:10.3389/fcell.2021.708715)
Supplement: Supplementary file 1 [file Table_1.DOCX]

Supplementary Material

# Supplementary Data

All supplementary data is available at Dryad Digital Repository system:

Supplementary movies:

https://datadryad.org/stash/share/GcyfEe1MBlUuy9I0Y23QbUFTbnbxnO6vXiLAGtuaFFs

TIRFM imaging dataset:

Microsoft Excel Spreadsheet includes a list of D values of every trajectory of GluR2-AMPA or mGluR1 of each group obtained from original videos. Dataset of Fig 1E, 1F, 2A, 2B, 2C, 2D, 3B, 5B1, 5B2, 5C1, 5C2, 6B, 6C.

https://datadryad.org/stash/share/CfpG51ymDCSqTNeWBgG8ZK2u9osZwFRaks0w4o0Srsk

STORM imaging dataset:

The dataset includes detailed STORM experimental results with original output files of the NIKON NIS-Elements imaging software containing the localization points for GluR2-AMPAR and GPER1 molecules (STORM_data.xlsx) as well as the corresponding confocal images (TIFF files).

<https://datadryad.org/stash/share/5UDMtN6CZQIJmC0e3s10zeMo9LfH6oaEv4PXMeZfRCs>

# Legends for Supplementary Figures and Movies

**Figure S1. Characterization of neuronal properties of differentiated PC12 cells.** ***A***, Differential interference contrast microscopy image depicts PC12 cells after 4 days of NGF treatment. ***B***, Immunofluorescence staining of microtubule-associated protein 2 (MAP2) and β-III tubulin shows the presence of neuronal markers. Scale bars: 20 µm. ***C,*** Left: immunofluorescence image of a biocytin loaded dPC12. Right: representative traces of the membrane potential in response to step current injection in dPC12. Scale bar: 20 µm.

**Figure S2. GPER1 distribution on dPC12 neurite**. The line graph of fluorescent intensity shows no GPER1 compartmentalization on representative neurites of dPC12. 100 nM E2 treatment does not GPER1 distribution.

**Figure S3. Viability test of dPC12**. A two-color fluorescence cell viability assay (LIVE/DEAD® Viability/Cytotoxicity Assay Kit, Thermofischer) was applied to detect the viability of dPC12. Photomicrographs depict live (green) and dead (red) dPC12 after different treatments. The viability assay demonstrates that dPC12 are alive in the medium and retained their viability and membrane integrity after 20 minutes 0.1% DMSO or 1 μM latA treatment (green dPC12). For positive control, 1% hydrogen peroxide (H_2_O_2_) treatment was used. H_2_O_2_ induced cell death in dPC12 (red dPC12). Scale bar: 20 µm, insert scale bar: 10 µm

**Figure S4. Validation of single-molecule labeling of hippocampal neurons.** Left, the intensity profile of a single ATTO 488-labeled GluR2-AMPAR signal on a hippocampal neuron. The arrow indicates single-step photobleaching. Right**,** Histogram showing the intensity value of every spot found in a recording of ATTO 488-labeled GluR2-AMPAR superimposed with a single fitted lognormal curve (blue line).

**Figure S5. The specificity of ATTO 488-labelled anti-GluR2-AMPAR and mGluR1 antibodies**. Left, Live-cell labeling of dPC12 neurites with ATTO 488-labeled anti-GluR2-AMPAR and mGluR1 antibodies. Right, No immunoreactivity was observed after pre-incubating with blocking peptides (control peptides) (GluA2_179-193_ peptide and mGluR1_501-516_ peptide, Alomone Labs). Scale bar: 2 µm

**Supplementary movie 1, 2.** Single molecules of ATTO 488-labeled GluR2-AMPAR, moving on the somas (1) and neurites (2) of live dPC12. The recordings were performed in TIRF mode with 34 ms acquisition time, and frames were displayed at 29 fps. Scale bar: 5 µm.

**Supplementary movie 3, 4.** Single molecules of ATTO 488-labeled mGluR1, moving on the somas (3) and neurites (4) of live dPC12. The recordings were performed in TIRF mode with 34 ms acquisition time, and frames were displayed at 29 fps. Scale bar: 5 µm.

**Supplementary movie 5**. Single molecules of ATTO 488-labeled GluR2-AMPAR moving on the neurite of live dPC12 cell after vehicle or 100 nM E2 treatment. The recordings were performed in TIRF mode with 34 ms acquisition time, and frames were displayed at 29 fps. Scale bar: 0.5 µm

**Supplementary movie 6.** Single molecules of ATTO 488-labeled AMPAR, moving on the neurites of a hippocampal neuron. The recording was performed in HILO mode with 34 ms acquisition time, and frames were displayed at 29 fps. Scale bar: 2 µm.

**Supplementary movie 7.** Single synaptic ATTO 488-labeled GluR2-AMPAR (green) at the synapse (red) labeled with MitoTracker Deep Red. The recording was performed in HILO mode with 36 ms acquisition time, and frames were displayed at 20 fps. Scale bar: 1 µm.

**Supplementary movie 8.** Single ATTO 488-labeled GluR2 subunits, moving on the membrane of a GluR2 subunit transfected CHO cell (overlayed to the corresponding differential interference contrast microscopy image). In non-transfected cells no ATTO 488 signal was detected. The recording was performed in TIRF mode with 34 ms acquisition time, and frames were displayed at 29 fps. Scale bar: 2 µm.

**Supplementary movie 9.** *In vivo* labeling with ATTO 488-conjugated anti-GluR2-AMPAR antibody showed no immunoreactivity on the soma of hippocampal neuron and detected moving GluR2-AMPAR molecules on neurites. The recording was performed in HILO mode with 34 ms acquisition time, and frames were displayed at 29 fps. Scale bar: 5 µm.

**
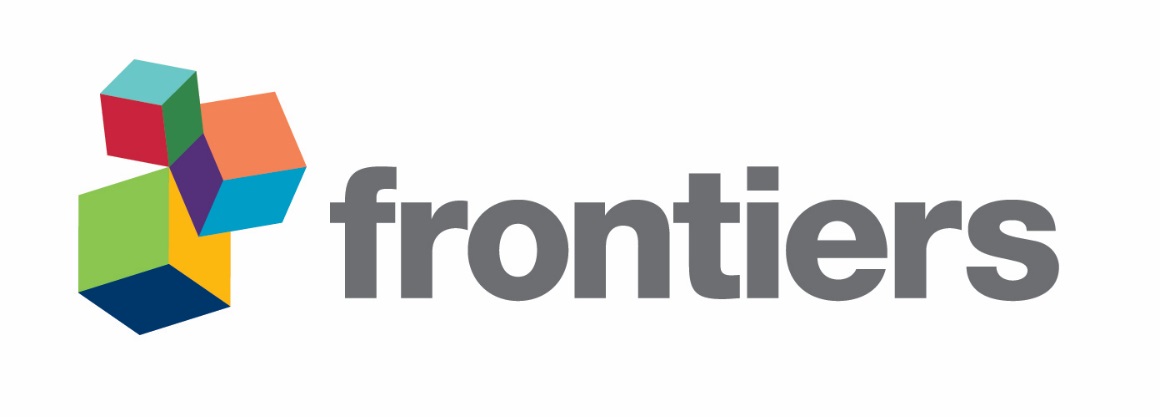
**
