# Supplementary figures and images for: Single-Molecule Imaging Reveals Rapid Estradiol Action on the Surface Movement of AMPA Receptors in Live Neurons
Source: Front Cell Dev Biol. 2021 Sep 23;9:708715. doi: 10.3389/fcell.2021.708715 (PMC8495425; doi:10.3389/fcell.2021.708715)

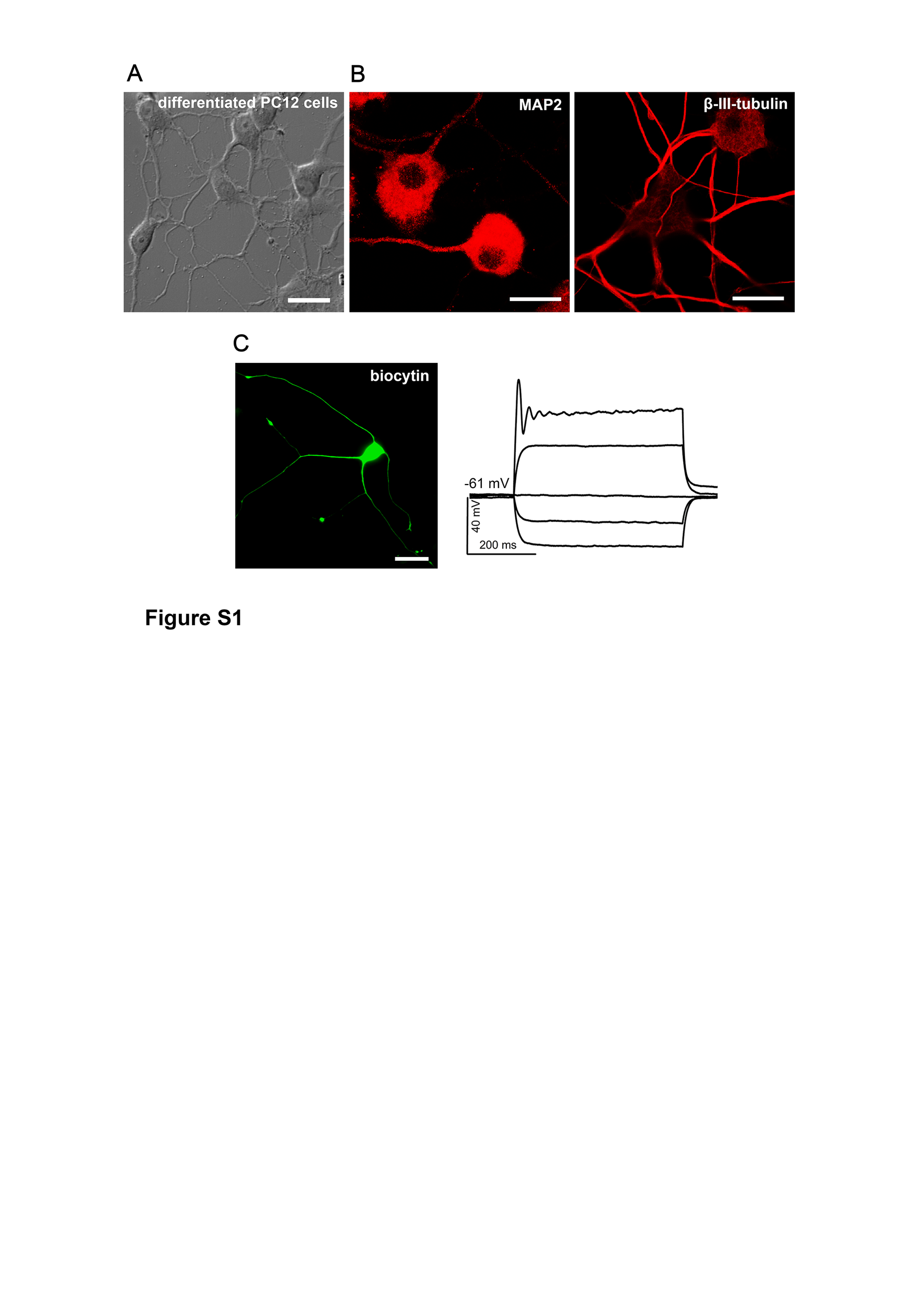

Supplement: Supplementary file 2 [file Image_1.TIF]

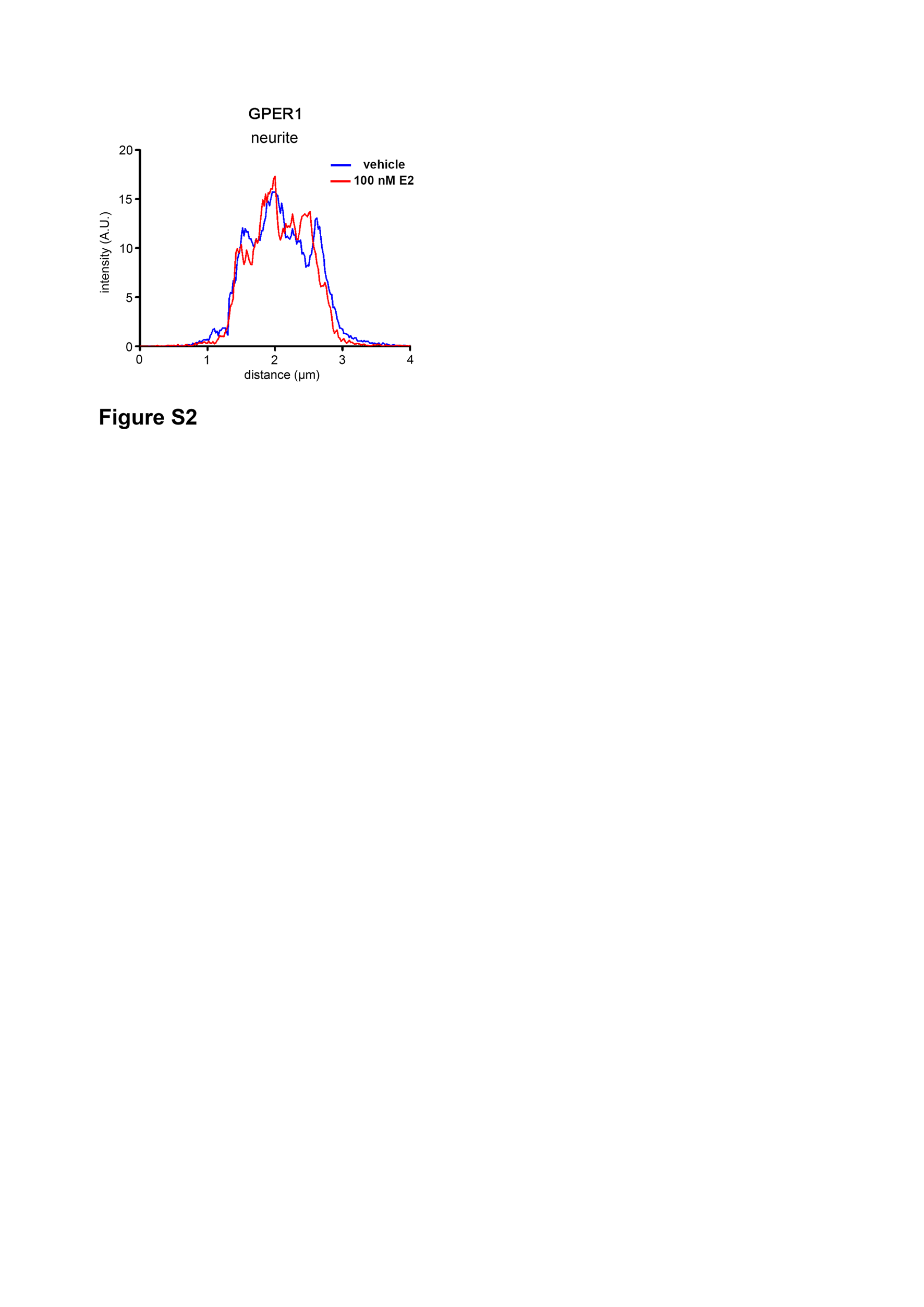

Supplement: Supplementary file 3 [file Image_2.TIF]

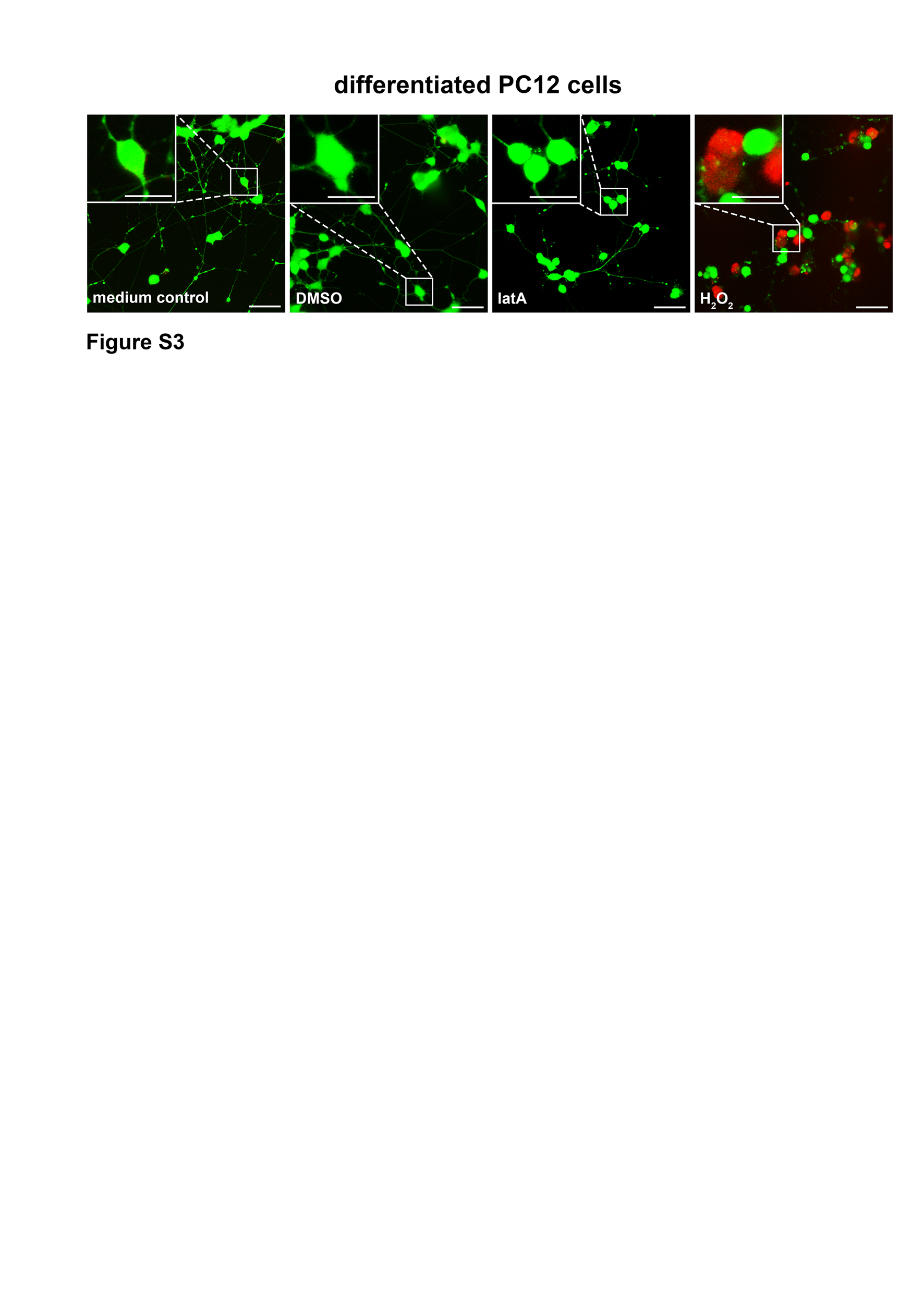

Supplement: Supplementary file 4 [file Image_3.TIF]

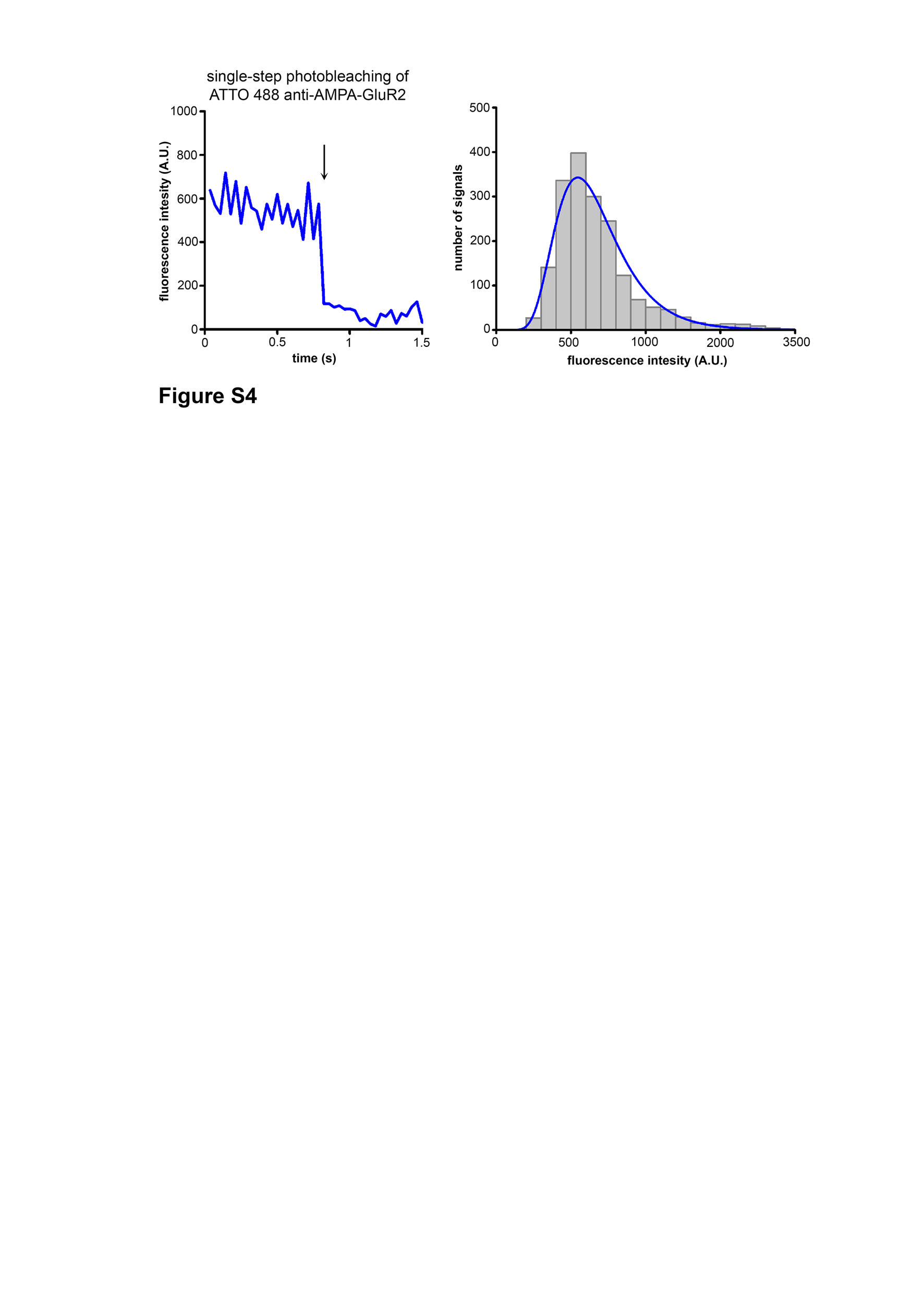

Supplement: Supplementary file 5 [file Image_4.TIF]

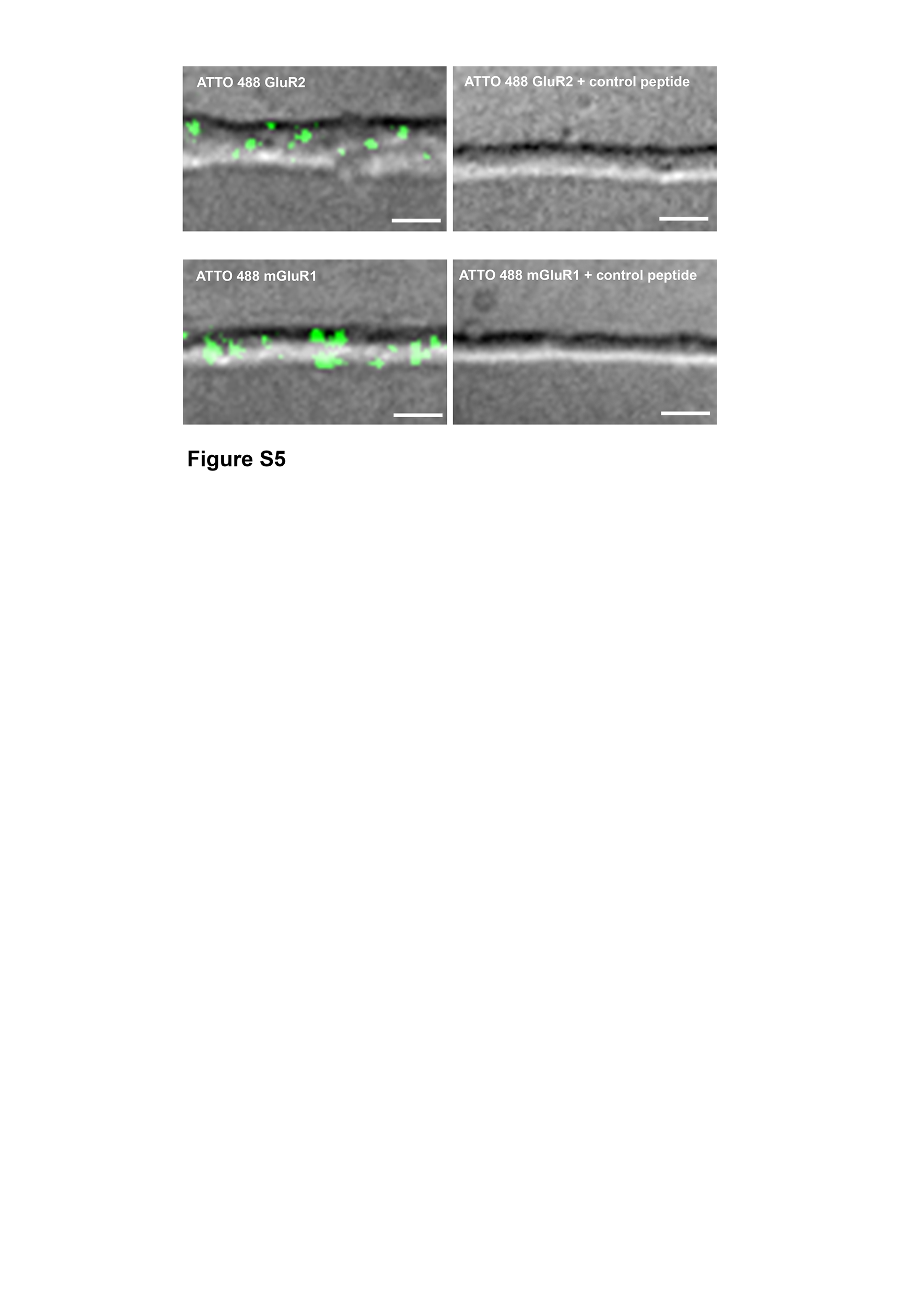

Supplement: Supplementary file 6 [file Image_5.TIF]
